# Studies toward the Total Synthesis of Itralamide B and Biological Evaluation of Its Structural Analogs

**DOI:** 10.3390/md13042085

**Published:** 2015-04-13

**Authors:** Xiaoji Wang, Chanshan Lv, Junmin Feng, Linjun Tang, Zhuo Wang, Yuqing Liu, Yi Meng, Tao Ye, Zhengshuang Xu

**Affiliations:** 1 School of Pharmacy, Jiangxi Science and Technology Normal University, Nanchang 330013, China; E-Mails: dashan10281985@126.com (C.L.); vonjimi@vip.qq.com (J.F.); linjun.tang@163.com (L.T.); 2 Laboratory of Chemical Genomics, Peking University Shenzhen Graduate School, University Town of Shenzhen, Xili, Nanshan District, Shenzhen 518055, China; E-Mail: mengy@pkusz.edu.cn; 3 Department of Applied Biology & Chemical Technology, The Hong Kong Polytechnic University, Hung Hom, Kowloon, Hong Kong; E-Mails: w.jasmine85@gmail.com (Z.W.); yuqing_liu@hotmail.com (Y.L.)

**Keywords:** itralamide B, cyclodepsipeptide, total synthesis, structural analogs, biological study

## Abstract

Itralamides A and B were isolated from the lipophilic extract of *Lyngbya majuscula* collected from the eastern Caribbean. Itralamide B (**1**) showed cytotoxic activity towards human embryonic kidney cells (HEK293, IC_50_ = 6 μM). Preliminary studies disapproved the proposed stereochemistry of itralamide. In this paper, we will provide a full account of the total synthesis of four stereoisomers of itralamide B and the results derived from biological tests of these structural congeners.

## 1. Introduction

**Figure 1:**
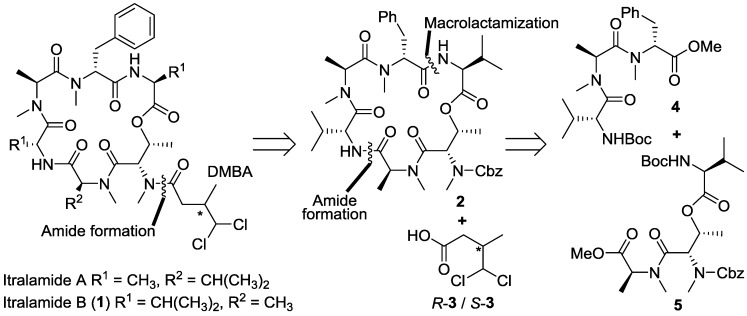
Structure of itralamides and the 1st retrosynthetic analysis of itralamide B (**1**).

Cyclodepsipeptide represents a classical sub-category of natural product, characterized by at least one ester bond embedded in the macrocycle. Natural cyclodepsipeptides often display intriguing biological activities, and some of them had been developed into lead compounds for further medicinal investigations [1]. In 2009, Horgen’s group reported the isolation of itralamides A and B **1** (Figure 1) from the lipophilic extract of *Lyngbya majuscula* [2]. Itralamides A and B are cyclodepsipeptides sharing the same 4,4-dichloro-3-methylbutanoic acid (DMBA) sidechain moiety with unknown stereochemical configuration. The macrocycle of itralamides is composed of a few *N*-methylated amino acids, including *N*-methyl threonine, which is not a conventional modification of amino acid for natural products [3,4]. We have been interested in the synthesis of bioactive natural cyclodepsipeptides [5,6,7,8,9,10,11], and successfully reassigned a few natural products [12,13,14,15,16]. In order to identify the absolute stereochemistry of the DMBA fragment presented in itralamides A & B, we conducted a synthetic study of itralamide B **1**. Our other objective was to possibly accelerate the structure-activity relationship studies of cytotoxic itralamide B regarding different cancer cell lines.

## 2. Results and Discussion

### 2.1. The First Generation Synthetic Endeavors

#### 2.1.1. Retrosynthetic Analysis

The absolute stereochemistry of the DMBA fragment must be determined via total synthesis, so in the first generation retrosynthetic analysis we detached this fragment from the macrocycle, producing the cyclodepsipeptide **2** and the DMBA fragment *R*-/*S*-**3** (Figure 1). This late stage introduction of the DMBA side-chain would allow the facile synthesis of different stereoisomers of itralamide B. The cyclic hexapeptide **2** could be constructed via the macrolactamization at the d-Phe/l-Val amide bond, and further disconnection at the l-MeAla/d-Val amide bond produced two tripeptides **4** and **5**. The above retrosynthetic strategy also provided an opportunity for the synthesis of **2** with an alternative fragment assembly, which led to the macrolactamization to be conducted at the l-MeAla/d-Val amide bond.

#### 2.1.2. Synthesis of Tripeptide **5**

**Scheme 1:**
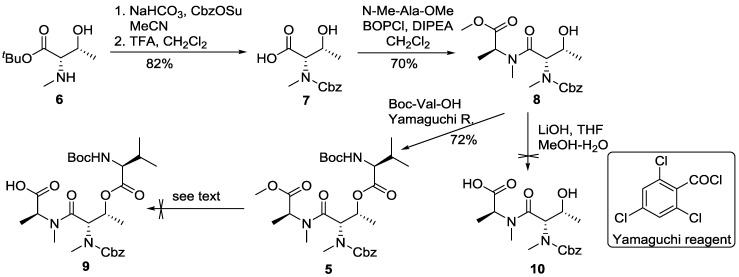
Attempted synthesis of fragment **9**.

The synthesis commenced with the treatment of **6** with CbzOSu and sodium bicarbonate in acetonitrile to protect the secondary amine. The *tert*-butyl ester was removed by the action of trifluoroacetic acid to produce carboxylic acid **7** at 82% yield. Condensation of **7** with *N*-Me-Ala-OMe was mediated by BOPCl [17,18] and DIPEA to give rise to dipeptide **8** at 70% yield. Esterification of **8** with Boc-Val-OH was facilitated by the modified Yamaguchi’s protocol [19,20], and depsipeptide **5** was obtained at 72% yield. To our surprise, selective removal of the methyl ester in **5** was found to be problematic. Different concentrations and equivalents of lithium hydroxide or sodium hydroxide as well as different solvent systems were applied to compound **5**, but produced no desired free acid **9**. The more selective method with lithium iodide in hot ethyl acetate also failed to provide any detectable quantities of product [21,22]. Although there were two ester bonds in **5**, we believed that the steric hindrance of the inner ester bond (Val-MeThr) was large enough to be differentiated from the terminal methyl ester. In fact, we did not find any fully hydrolyzed dipeptide **10** from the reaction mixture (Scheme 1). 

When dipeptide **8** was subjected to hydrolysis with lithium hydroxide under standard reaction conditions, we could not isolate the desired acid **10** either. The major product of this saponification reaction, although not fully characterized, revealed the Cbz group was cleaved. The failure to remove the methyl ester was assumed to be due to interference by the Cbz group at threonine; the *N*-methylation of threonine changed the conformation of the peptide and thus promoted side reactions.

Because the preparation of tripeptide **9** was unsuccessful, we decided to form an amide bond on the nitrogen of threonine instead of protecting it with Cbz, which could mimic the natural product structure and allow us to examine the feasibility of macrolactamization as illustrated in Figure 1.

#### 2.1.3. Model Study for Macrolactamization at Different Amide Bonds

**Scheme 2:**
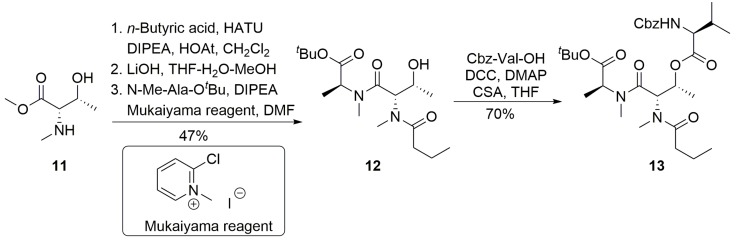
Preparation of tetrapeptide **13**.

As shown in Scheme 2, *n*-butyric acid derived amide was elected to mimic the DMBA fragment. Thus, *N*-Me-Thr-OMe **11** was condensed with *n*-butyric acid in the presence of HATU and HOAt to give rise to the corresponding dipeptide. The methyl ester was smoothly hydrolyzed with lithium hydroxide, and the resultant dipeptide acid was then coupled to N-Me-Ala-O*^t^*Bu using Mukaiyama reagent [23,24] to produce tripeptide **12** at 47% yield over three steps (Scheme 2). Esterification of **12** with *N*-Cbz-Val-OH was facilitated by the modified Keck condition [25], in the presence of DCC, DMAP and a catalytic amount of CSA, and depsipeptide **13** was prepared at 70% yield.

**Scheme 3:**
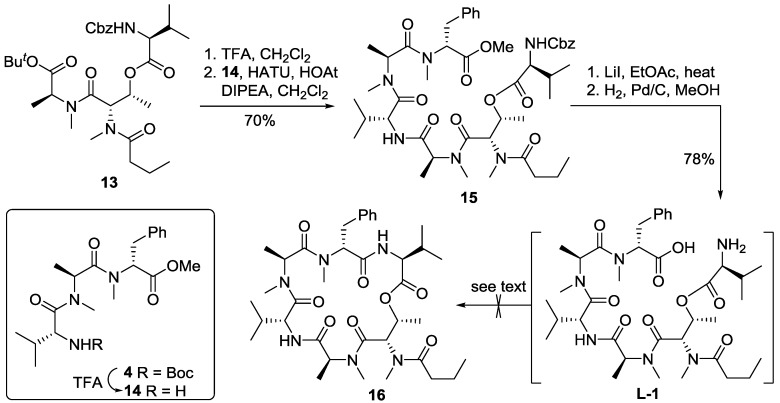
Attempts at macrolactamization at the Phe-Val site.

Further elaboration of depsipeptide **13** is illustrated in Scheme 3. Thus, acidic cleavage of the *tert*-butyl ester of **13** and the Boc group in **4** [26] afforded the corresponding acid and TFA salt of amine **14**, respectively. Coupling of these two fragments was carried out with HATU and HOAt in dichloromethane to produce heptapeptide **15** in 70% yield. The methyl ester was then cleaved via a S_N_2-type saponification process mediated by heating a solution of **15** and lithium iodide in ethyl acetate [21,22]. Subsequent hydrogenolytic removal of the Cbz group produced the linear precursor **L-1** in 78% yield over two steps. The macrolactamization of **L-1** was performed in the presence of PyAOP [27], HATU or Mukaiyama reagent under various conditions (using DMF or MeCN as solvent and different reaction temperatures). Unfortunately and notwithstanding this progress, all of our attempts failed to provide desired product **16** (Scheme 3), presumably due to a conformational disposition of the linear precursor that prevented the macrocyclization.

**Scheme 4:**
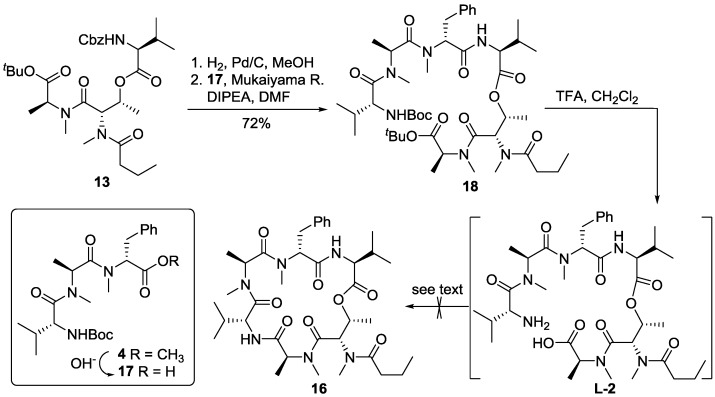
Attempts for macrolactamization at the MeAla-Val site.

With both coupling partners **13** and **4** in hand, we decided to carry out the macrolactamization at the MeAla-Val site. Thus, removal of the Cbz group of **13** afforded the corresponding amine, which was then coupled with acid **17**, prepared by basic hydrolysis of **4**, and proceeded smoothly to give heptapeptide **18** at 72% yield. Concomitant removal of the *tert*-butyl ester and Boc group of **18** was carried out using trifluoroacetic acid in dichloromethane to produce the linear precursor **L-2**, which was subjected to macrocyclization in identical conditions as those described for **L-1**. To our disappointment and surprise, all attempts to effect the macrolactamization were unsuccessful and no desired product was isolated (Scheme 4). 

**Figure 2:**
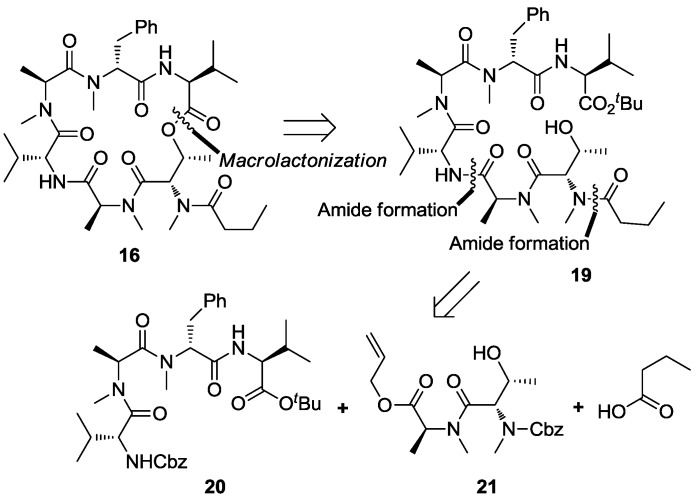
Revised retrosynthetic analysis for model study.

In general, macrolactonization of peptide-containing hydroxy acids is a more difficult task than similar amide bond-forming cyclizations. Given the fact that two approaches based on macrolactamization did not lead to the formation of macrocycle **16**, it was considered necessary at this stage to investigate the alternative yet unprecedented macrolactonization [28] route as depicted, in a retrosynthetic format (Figure 2).

#### 2.1.4. Model Study for Macrolactonization

The ester bond between valine and threonine was selected as the macrocyclization site. The linear precursor **19** was disconnected into tetrapeptide **20**, dipeptide **21** and *n*-butyric acid. In order to circumvent the problem associated with saponification of the methyl ester of dipeptide MeThr-MeAla, the carboxylic acid terminus of dipeptide **21** was protected as its allyl ester, which could be readily removed via a palladium catalyzed process [29].

**Scheme 5:**
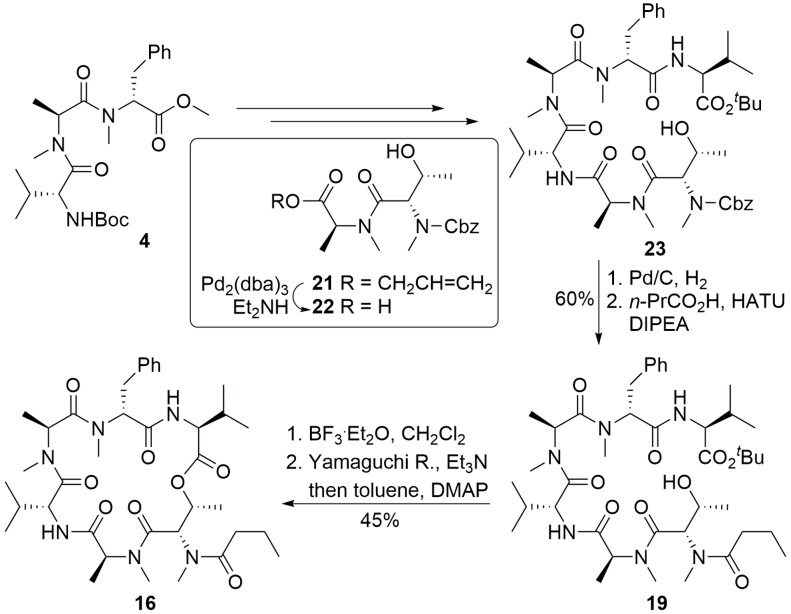
Model study for macrolactonization.

Tripeptide **4** was transformed into hexapeptide **23** according to the procedure described in our previous communication [26]. Hydrogenolytic removal of the *N*-terminal Cbz followed via a condensation of the resultant free amine with *n*-butyric acid through the action of HATU and HOAt to produce heptapeptide **19** at 60% yield. Treatment of **19** with boron trifluoride etherate [30] in dichloromethane smoothly liberated the carboxylic acid, and macrolactonization using the Yamaguchi protocol produced the desired cyclodepsipeptide **16** at 45% isolable yield over two steps (Scheme 5).

### 2.2. Synthesis of Different Stereoisomers of Itralamide B

#### 2.2.1. Completion of Total Synthesis of Itralamide B **1a** and **1b**

**Scheme 6:**
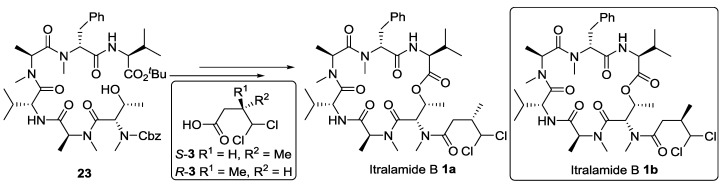
Completion of the total synthesis of itralamide B **1a** and **1b**.

Encouraged by the success of this model study that yielded a compound **16** closely related to the itralamide B target molecule, we proceeded with a synthesis of two diastereoisomers of itralamide B (**1a** and **1b**) including the side chain attachment [26]. This was readily achieved by following the same synthetic procedure as for **16**, but using either *S*-**3** or *R*-**3** instead of *n*-butyric acid (Scheme 6).

**Figure 3:**
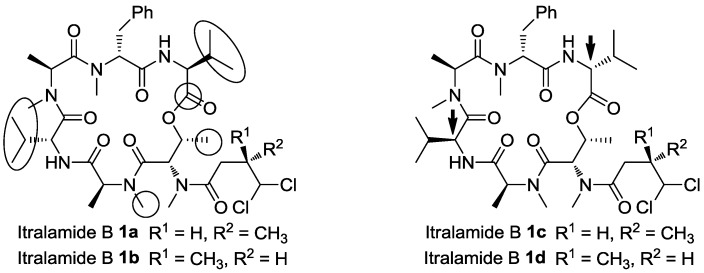
Analysis of the stereochemistry.

**Figure 4:**
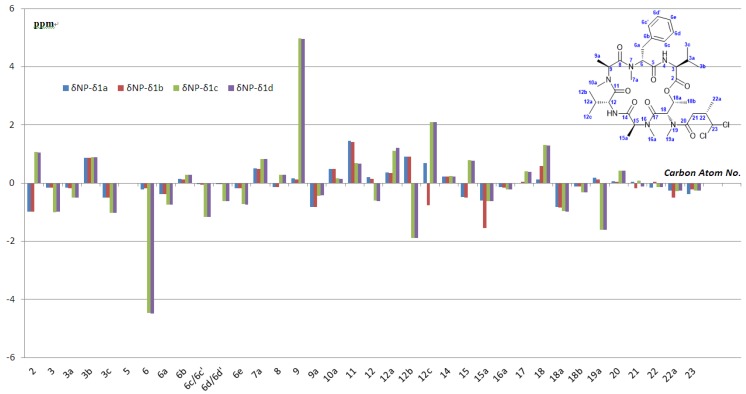
Comparison of the ^13^C NMR data.

On examining the analytical information, we were disappointed as the authentic data did not match those of our products **1a** and **1b** [2]. There were significant discrepancies in the chemical shifts in the region closed to these two valine residues, particularly the ^13^C NMR chemical shifts at the *iso*-propyl groups of two valines, the two *N*-methyl groups of methylalanines, the methyl group of threonine and the ester carbonyl (Figure 3; see also Figure 4 and the Supplementary Information for intuitionistic comparison). According to the isolation paper, the macrocyclic structure and connectivity of itralamide B were established by NMR studies, and the absolute configuration of amino acids was determined by Marfey’s advanced analytical method [31]. Since itralamide B contains two valine residues with the opposite configuration, their respective assignments remained uncertain. Although the stereochemistry of the macrocyclic core was assumed to be that shown in Figure 1, the issue related to the absolute configuration of each valine was left largely unresolved in the original isolation paper. The synthesis of **1a** and **1b** has disproved the original assigned structure for itralamide B. Therefore, we hypothesized that the incorrect structure proposed for itralamide B could possibly be a result of misassignment of the absolute configuration of two valine residues. We therefore elected to synthesize two diastereomers (**1c** and **1d**, Figure 3) of the proposed structure. 

#### 2.2.2. Synthesis of Two Additional Stereoisomers of Itralamide B **1c** and **1d**

**Scheme 7:**
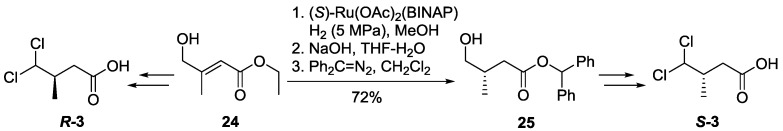
Improved synthesis of DMBA fragment **3**.

Prior to embarking on the synthesis of **1c** and **1d**, a more efficient route to **3** was then developed from the known ester **24** [32] (Scheme 7).

Thus, hydrogenation of the known unsaturated ester **24** with commercially available chiral catalyst (*S*)-Ru(OAc)_2_(BINAP) [33] produced the corresponding saturated ester at high yield with 93% enantioselectivity (ee). After saponification, the resultant acid was reacted with diphenyldiazomethane [34] in dichloromethane to give rise to the benzhydryl ester **25** at 72% yield over three steps. Subsequent conversion of alcohol **25** to carboxylic acid *S-***3** was performed according to reported procedures [26]. In addition, *R*-**3** was prepared by following the same synthetic procedure as for *S*-**3**, but using (*R*)-Ru(OAc)_2_(BINAP) as the catalyst.

**Scheme 8:**
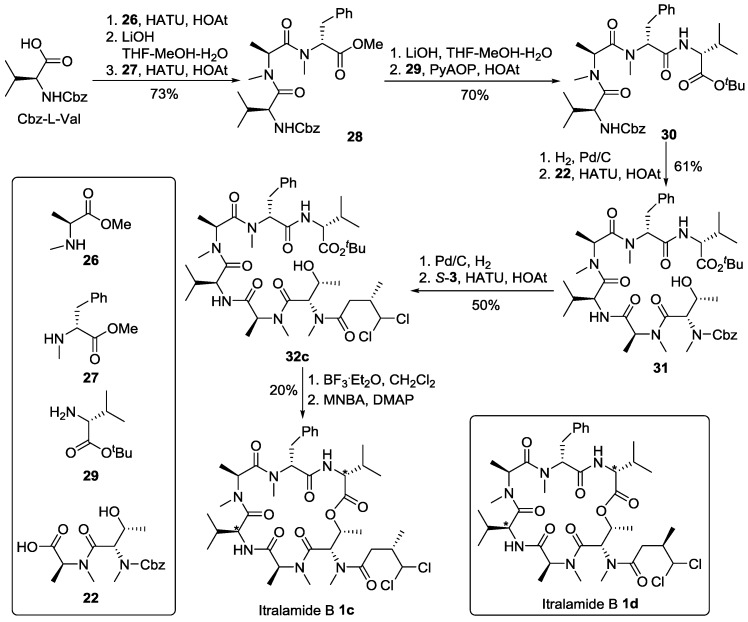
Synthesis of stereoisomers itralamide B **1c** and **1d**.

As outlined in Scheme 8, the synthesis of itralamide B **1c** and **1d** commenced with the coupling of Cbz-l-Val with *N*-Me-Ala-OMe (**26**) using HATU and HOAt as dehydration reagents to produce the corresponding dipeptide. Subsequent saponification and HATU/HOAt-mediated coupling to *N*-Me-Phe-OMe (**27**) provided tripeptide **28** at 73% yield. Hydrolysis of the methyl ester with lithium hydroxide was followed by coupling with d-Val-O*^t^*Bu (**29**), promoted by PyAOP and HOAt to produce tetrapeptide **30** at 70% yield. Hydrogenolysis of the Cbz group of **30** using palladium on charcoal smoothly produced the correponding free amine, which was then condensed with dipeptide acid **22** in the presence of HATU and HOAt to generate hexapeptide **31** at 61% yield. Hexapeptide **31** was further elaborated to the linear precursor **32c** using a two-step sequence involving hydrogenolysis of the Cbz group and subsequent HATU/HOAt-mediated fragment condensation of the resultant amine with DMBA fragment *S*-**3**. Treatment of **32c** with boron trifluoride etherate in dichloromethane, followed by macrolactonization using the Shiina reagent (2-methyl-6-nitrobenzoic anhydride) [35] produced itralamide B **1c** at 20% yield. Other macrolactonization protocols, such as the standard Yamaguchi macrolactonization conditions, led to substantially lower yields. Similarly, the DMBA fragment *R*-**3** was readily incorporated into the synthesis as previously performed to afford itralamide B **1d** with 11% overall yield from hexapeptide **31**. 

The NMR spectroscopic data (Figure 4) and optical rotation of these synthetic samples (**1a**–**1d**) are quite different from those of natural itralamide B. Similar to itralamide B **1a**,**b**, the diastereoisomers **1c** and **1d** showed significant discrepancies with the natural product on ^13^C NMR spectra at the *iso*-propyl groups of the valines, the *N*-methyl group of threonine and the ester carbonyl; the biggest differences (larger than 4 ppm) appeared at the α-stereogenic centers of MePhe (C6) and one of the MeAla (C9). The discrepancies remain unresolved issues which are subject to conjecture. 

### 2.3. Biological Study of Itralamide B and Structural Analogs

We next carried out the biological evaluation of our synthetic itralamide B **1a**–**1d** and the cyclodepsipeptide **16**. The inhibitory activity towards cell proliferation was evaluated by 3-(4,5-dimethylthiazol-2-yl)-5-(3-carboxymethoxyphenyl)-2-(4-sulfophenyl-2*H*-tetrazolium) (MTS) assay in four cancer cell lines (Table 1).

**Table 1:** The effect of compounds on the proliferation of cancer cell lines.

*Origins*	*Cell Line*	Inhibitory Activity (IC_50_ in μM)					
		*Compound 16*	*Itralamide B 1a* (RSD)	*Itralamide B 1b*	*Itralamide B 1c* (RSD)	*Itralamide B 1d* (RSD)	*Largazole ^a^* (RSD)
Brain	SH-SY5Y	-	-	-	56.8 (2.3%)	56.4 (4.1%)	0.8 (1.1%)
Cervix	HeLa	-	-	-	38.0 (4.6%)	82.5 (4.3%)	2.8 (1.7%)
Liver	Hep3B	-	97.8 (1.4%)	-	-	-	0.3 (3.4%)
	PLC	-	33.1 (2.8%)	-	34.3 (3.4%)	39.5 (3.8%)	0.8 (2.5%)

*^a^* Positive control.

Compound **16** and itralamide B **1b** showed no inhibitory effect towards four cancer cell lines, while itralamide **1a** exerted some inhibitory effect against PLC cells with IC_50_ of 33.1 µM. Futhermore, itralamide B **1c** and **1d** were found to be more active than itralamide **1a** and **1b**. Presumably this may be attributed to the conformation change derived from the inversion of the configuration of valine residue presented in the macrocycle. Itralamide B **1c** inhibited the proliferation of HeLa and PLC/RPF/5 (PLC) in a dose-dependent manner with IC_50_ of 38.0 µM and 34.3 µM, respectively. Itralamide B **1d** exhibited dose-dependent inhibitory effect against cell proliferation of PLC with IC_50_ of 39.5 µM.

## 3. Experimental Section

### 3.1. General Experimental

All non-aqueous reactions were run under a nitrogen or argon atmosphere and all reaction vessels were oven-dried. Solvents were distilled prior to use: tetrahydrofuran (THF) from Na/benzophenone, dichloromethane (DCM), triethylamine and diisopropylethylamine (DIPEA) from CaH_2_. NMR spectra were recorded on Bruker spectrometers. Chemical shifts were reported in parts per million (ppm), relative to the signals due to the solvent. Data were reported as follows: chemical shift, multiplicity (s = singlet, d = doublet, t = triplet, q = quartet, br = broad), integration and coupling constants. Some peptide intermediates exist as rotamers due to N-methylation and the increased steric hinderence, their chemical shifts for the minor isomer on ^1^H NMR spectra were recorded in parentheses next to the major isomer, while for ^13^C NMR the chemical shifts were recorded as they were and not differentiated. ESI mass spectra were obtained using a Finnigan MAT 95 mass spectrometer. Optical rotations were recorded on a Perkin Elmer 343 Polarimeter. TLC were carried out using pre-coated sheets (Qingdao silica gel 60-F_250_, 0.2 mm, Qingdao, China) and visualized at 254 nm, and/or staining in ninhydrin or phosphomolybdic acid solution followed by heating. Flash column chromatography was performed using the indicated solvents on E. Qingdao silica gel 60 (230–400 mesh ASTM).

### 3.2. Synthesis of Cyclodepsipeptide **16** and Itralamide B **1c** and **1d**

#### 3.2.1. Preparation of Cyclodepsipeptide **16**

To a solution of compound **23** (200.0 mg, 0.23 mmol) in MeOH (20 mL), Pd/C was added under N_2_ atmosphere. The reaction vessel was sealed and flashed with H_2_ three times. The reaction mixture was then vigorously stirred overnight under H_2_ atmosphere. Catalyst residue was removed by filtration. The filtrate was concentrated *in vacuo* to give the corresponding free amine, which was pure enough and used directly in the next step of reaction. To a solution of *n*-PrCO_2_H (60.0 mg, 0.69 mmol) in DCM (2 mL), HATU (175.0 mg, 0.46 mmol) was added, followed by addition of DIPEA (200 μL, 1.15 mmol) at 0 °C. 0.5 h later, a solution of above amine (72.0 mg, 0.23 mmol) in DCM (2 mL) was addedat 0 °C. The reaction mixture was allowed to warm to room temperature and stirred overnight. The reaction was quenched with saturated NH_4_Cl (20 mL), and extracted with DCM (3 × 50 mL). The combined organic layers were washed with saturated NaHCO_3_ solution (3 × 50 mL) and brine (50 mL), dried over anhydrous Na_2_SO_4_ and concentrated in *vacuo*. The residue was purified by flash chromatography (ethyl acetate) to give compound **19** (109.0 mg, 60%). [*α*]_D_^25^ = −45.3 (*c* 0.5, CHCl_3_); ^1^H NMR (400 MHz, CDCl_3_) δ 7.25–7.15 (m, 5H), 6.71 (d, *J* = 8.9 Hz, 1H), 6.64 (d, *J* = 8.6 Hz, 1H), 5.43–5.41 (m, 1H), 5.17–5.08 (m, 1H), 4.66 (dd, *J* = 8.8, 6.3 Hz, 1H), 4.39 (dd, *J* = 6.5, 3.6 Hz, 1H), 4.34 (dd, *J* = 8.5, 4.8 Hz, 1H), 4.20 (d, *J* = 3.3 Hz, 1H), 3.37 (dd, *J* = 14.4, 7.4 Hz, 1H), 3.06 (s, 3H), 2.93 (s, 3H), 2.90–2.85 (m, 2H), 2.86 (s, 3H), 2.75 (s, 3H), 2.44–2.34 (m, 2H), 2.14–2.06 (m, 1H), 2.02–1.97 (m, 1H), 1.78−1.65 (m, 2H), 1.46 (s, 9H), 1.35 (d, *J* = 7.2 Hz, 3H), 1.15 (d, *J* = 6.4 Hz, 3H), 1.06 (d, *J* = 6.9 Hz, 3H), 0.99 (d, *J* = 5.1 Hz, 3H), 0.93 (d, *J* = 6.7 Hz, 3H), 0.87 (d, *J* = 6.8 Hz, 3H), 0.83 (d, *J* = 6.9 Hz, 3H), 0.82 (d, *J* = 6.9 Hz, 3H). ^13^C NMR (100 MHz, CDCl_3_) δ 173.7, 172.6, 172.0, 171.4, 170.8, 170.3, 169.4, 136.9, 129.0, 128.4, 126.6, 81.8, 68.1, 57.7, 56.8, 54.2, 52.5, 50.1, 38.6, 35.2, 34.0, 33.5, 31.0, 30.8, 30.7, 30.5, 28.0, 19.6, 18.9, 18.8, 18.5, 17.7, 17.4, 14.3, 13.9, 13.7. HR-ESIMS *m/z* for C_41_H_68_N_6_NaO_9_^+^ [M + Na]^+^: calculated 811.4940, found 811.4941.

To a solution of compound **19** (23.0 mg, 0.03 mmol) in DCM (1.0 mL), BF_3_^.^Et_2_O (38 μL, 0.3 mmol) was added dropwise at 0 °C. The reaction solution was then allowed to warm to room temperature and stirred for 0.5~1.0 h (monitored by TLC). The reaction was quenched by addition of saturated NH_4_Cl (2 mL) and diluted with DCM (60 mL). The organic phase was washed with saturated NH_4_Cl (3 × 20 mL) and brine (20 mL), dried over anhydrous Na_2_SO_4_ and concentrated in *vacuo* to produce crude hydroxy acid, which was dried further under high vacuum for 4 h. To a solution of the above acid (50.0 mg, 0.07 mmol) in THF (5 mL) was added Et_3_N (59 μL, 0.41 mmol) and trichlorobenzoyl chloride (54 μL, 0.34 mmol). The reaction mixture was stirred at room temperature for 3 h and diluted with toluene (3 mL). The resulted solution was added to a solution of DMAP (208.2 mg, 1.71 mmol) in toluene (50 mL) via a syringe pump over 48 h at 30 °C. The reaction was concentrated *in vacuo*, and the residue was dissolved in ethyl acetate (80 mL) and washed with saturated ammonium chloride (100 mL). Layers were separated, and the aqueous phase was extracted with ethyl acetate (2 × 80 mL). The combined organic layers were washed with brine (80 mL), dried over sodium sulfate and concentrated *in vacuo*. The residue was purified by flash chromatography (ethyl acetate) to give compound **16** (9.5 mg, 45%) as a yellow oil. [*α*]_D_^25^ = −65.4 (*c* 0.6, CHCl_3_); ^1^H NMR (400 MHz, CDCl_3_) δ 7.24–7.16 (m, 5H), 6.89 (d, *J* = 9.6 Hz, 1H), 6.48 (d, *J* = 7.9 Hz, 1H), 5.77 (d, *J* = 3.2 Hz, 1H), 5.70 (dd, *J* = 12.3, 4.8 Hz, 1H), 5.47 (dd, *J* = 6.6, 3.2 Hz, 1H), 5.08 (q, *J* = 6.9 Hz, 1H), 4.98 (dd, *J* = 7.9, 4.3 Hz, 1H), 4.70 (dd, *J* = 9.4, 4.1 Hz, 1H), 4.66–4.58 (m, 1H), 3.81 (t, *J* = 7.8 Hz, 1H), 3.66 (dd, *J* = 15.3, 5.2 Hz, 1H), 3.33 (s, 3H), 3.19 (3.18) (s, 3H), 3.16 (s, 3H), 3.02 (s, 3H), 2.39–2.35 (m, 2H), 1.45–1.37 (m, 2H), 1.30 (d, *J* = 7.2 Hz, 3H), 1.07–1.04 (m, 3H), 0.99–0.79 (m, 18H). ^13^C NMR (100 MHz, CDCl_3_) δ 174.9, 172.9, 172.1, 170.7, 170.2, 170.0, 169.8, 137.4, 128.6, 128.3, 126.5, 69.8, 57.0, 56.8, 54.4, 54.0, 51.4, 50.3, 35.2, 33.8, 33.8, 32.2, 31.8, 31.1, 31.0, 30.5, 22.7, 19.9, 19.6, 18.6, 18.3, 17.8, 17.1, 17.1, 13.8. HR-ESIMS *m/z* calculated for C_37_H_59_N_6_O_8_^+^ [M + H]^+^: 715.4389, found 715.4390.

#### 3.2.2. Preparation of Ester **25**

In a stainless steel autoclave, ester **24** (461.4 mg, 3.19 mmol) was dissolved in methanol (50 mL), after catalyst (*S*)-Ru(OAc)_2_(BINAP) (48.0 mg, 0.06 mmol) was added, the reaction mixture was stirred under hydrogen atmosphere (5 MPa) for 24 h. The organic solution was transferred to a round bottom flask and concentrated to 5 mL, and THF–H_2_O (10 mL, 1:1) was added, followed by addition of aqueous sodium hydroxide (6.4 mL, 6.4 mmol, 1 N in water). The solution was then stirred at room temperature for 12 h. Volatiles were removed under vacuum. The aqueous layer was extracted with diethyl ether (2 × 30 mL), and the organic solution was discarded. The aqueous solution was acidified to pH 3 with dilute hydrochloric acid (1 N in water) and extracted with dichloromethane (3 × 30 mL). The combined organic layers were dried over Na_2_SO_4_, filtered and concentrated in *vacuo* to 10 mL. Without further purifications, to the above organic solution at 0 °C, diphenyldiazomethane (0.71 g, 3.66 mmol) in dichloromethane (3 mL) was added. The reaction mixure was stirred for an additional 6 h and then concentrated *in vacuo*. The residue was purified using flash chromatography (ethyl acetate/hexane, 1:3) to provide **25** [26] as a yellow oil (653.4 mg, 72%).

#### 3.2.3. Preparation of Tripeptide **28**

To a solution of Cbz-l-Val (9.20 g, 36.64 mmol) and amine **26** (4.31 g, 28.18 mmol) in DCM (250 mL), HATU (21.43 g, 56.36 mmol), DIPEA (23.3 mL, 140.90 mmol) and HOAt (7.67 g, 56.36 mmol) were added sequentially at 0 °C. The reaction mixture was allowed to warm to room temperature and stirred overnight, then quenched by addition of saturated NH_4_Cl solution (200 mL) and extracted with DCM (3 × 80 mL). The combined organic layers were washed with saturated NaHCO_3_ solution (3 × 80 mL) and brine (80 mL), dried over anhydrous Na_2_SO_4_ and concentrated in *vacuo*_._ The residue was purified by flash chromatography (hexane/ethyl acetate, 1:1) to afford dipeptide Cbz-Val-MeAla-OMe (6.27 g, 64%). [α]_D_^20^ = −21.5, (*c* 1.1, CHCl_3_); ^1^H NMR (500 MHz, CDCl_3_) δ 7.40–7.25 (m, 5H), 5.59 (d, *J* = 7.5 Hz, 1H), 5.27 (q, *J* = 7.1 Hz, 1H), 5.15–5.04 (m, 2H), 4.55 (dd, *J* = 9.2, 5.9 Hz, 1H), 3.70 (s, 3H), 3.03 (2.84) (s, 3H), 2.10–2.00 (m, 1H), 1.41 (d, *J* = 7.4 Hz, 3H), 1.03 (d, *J* = 6.7 Hz, 3H), 1.00–0.87 (m, 3H). ^13^C NMR (75 MHz, CDCl_3_) δ 172.3, 172.0, 156.5, 136.4, 128.5, 128.1, 128.0, 66.9, 55.8, 52.2, 52.1, 31.3, 31.3, 19.4, 17.2, 14.1.

To a solution of Cbz-Val-MeAla-OMe (4.27 g, 12.19 mmol) in THF-MeOH-H_2_O (90 mL, 1:1:1) was added LiOH^.^H_2_O (1.46 g, 60.93 mmol) at 0 °C. The reaction mixture was allowed to warm to room temperature and stirred for 5 h (monitored by TLC). Volatiles were removed *in vacuo*, the aqueous solution was washed with Et_2_O (3 × 80 mL). The organic phases were discarded, and the aqueous phase was acidified to pH 3 with 10% aqueous solution of citric acid at 0 °C. The aqueous layer was extracted with ethyl acetate (3 × 80 mL). The combined organic layers were washed with brine (50 mL), dried over anhydrous Na_2_SO_4_, filtered and concentrated in *vacuo* to give the corresponding acid (4.10 g, 99%). This acid (4.10 g, 12.18 mmol), without further purification, was mixed with amine **27** (3.63 g, 15.84 mmol) and dissolved in DCM (80 mL) at 0 °C. To this solution, HATU (9.27 g, 24.38 mmol), DIPEA (10.1 mL, 60.95 mmol) and HOAt (3.32 g, 24.38 mmol) were added at 0 °C. The reaction mixture was then allowed to warm to room temperature and stirred for 16 h. The reaction was quenched with saturated NH_4_Cl solution (200 mL). Layers were separated, the aqueous layer was extracted with DCM (3 × 80 mL). The combined organic layers were washed with saturated NaHCO_3_ solution (3 × 80 mL) and brine (80 mL), dried over anhydrous Na_2_SO_4_ and concentrated in *vacuo*. The residue was purified by flash chromatography (hexane/ethyl acetate, 1:1) to afford tripeptide **28** (4.36 g, 70%) as a viscous oil. [α]_D_^25^ = +85.8 (*c* 2.3, CHCl_3_); ^1^H NMR (500 MHz, CDCl_3_) δ 7.39–7.25 (m, 7H), 7.24–7.10 (m, 3H), 5.59–5.45 (m, 1H), 5.43–5.13 (m, 2H), 5.12–4.82 (m, 2H), 4.51–4.25 (m, 1H), 3.73 (3.54) (s, 3H), 3.45–3.25 (m, 1H), 2.95–2.87 (m, 1H), 2.85 (2.82) (s, 3H), 2.61 (2.29) (s, 3H), 2.00–1.85 (m, 1H), 1.25–0.82 (m, 9H). ^13^C NMR (125 MHz, CDCl_3_) δ 171.7, 171.5, 171.0, 170.9, 170.8, 170.5, 170.4, 156.2, 136.9, 136.8, 136.4, 129.0, 129.0, 128.8, 128.7, 128.5, 128.1, 128.0, 128.0, 127.9, 127.0, 126.8, 66.9, 66.8, 66.8, 60.4, 60.4, 59.0, 58.3, 55.9, 55.6, 55.5, 52.5, 52.3, 49.8, 48.4, 35.1, 34.5, 34.4, 32.3, 32.3, 31.6, 31.2, 31.0, 30.0, 29.9, 29.3, 21.0, 19.8, 19.4, 19.2, 17.6, 17.1, 16.8, 14.3, 14.2, 14.0; HR-ESIMS *m/z* for C_28_H_38_N_3_O_6_^+^ [M + H]^+^: calculated 512.2755, found 512.2755.

#### 3.2.4. Preparation of Tetrapeptide **30**

To a solution of the tripeptide **28** (1.00 g, 1.95 mmol) in THF–MeOH–H_2_O (30 mL, 1:1:1) was added LiOH^.^H_2_O (250.0 mg, 5.94 mmol) at 0 °C. The reaction mixture was allowed to warm to room temperature and stirred for 5 h (monitored by TLC). Volatiles were removed *in vacuo*. The aqueous layer was washed with Et_2_O (3 × 80 mL). The organic phases were discarded, and the aqueous phase was acidified to pH 3 with 10% aqueous solution of citric acid at 0 °C. This aqueous layer was then extracted with ethyl acetate (3 × 80 mL). The combined organic layers were washed with brine (50 mL), dried over anhydrous Na_2_SO_4_, filtered and concentrated *in vacuo* to give the corresponding acid (850.0 mg, 87%). To a solution of above acid in DCM (50 mL), PyAOP (1.78 g, 3.42 mmol), DIPEA (1.4 mL, 8.55 mmol) and HOAt (470.0 mg, 3.42 mmol) were sequentially added at 0 °C. 0.5 h later, a solution of Val-O*^t^*Bu **29** (360.0 mg, 2.05 mmol) in DCM (5 mL) was added at 0 °C. The reaction mixture was allowed to warm to room temperature and stirred for 16 h. The reaction was quenched by addition of saturated NH_4_Cl solution (150 mL). Layers were separated, the aqueous phase was extracted with DCM (3 × 80 mL). The combined organic layers were washed with saturated NaHCO_3_ solution (3 × 50 mL) and brine (30 mL), dried over anhydrous Na_2_SO_4_ and concentrated *in vacuo*. The residue was purified by flash chromatography (hexane/ethyl acetate, 1:1) to afford tetrapepetide **30** (890.0 mg, 80%). [α]_D_^25^ = +32.5 (*c* 1.7, CHCl_3_); ^1^H NMR (500 MHz, CDCl_3_) δ 7.39–7.11 (m, 10H), 6.47–6.12 (m, 1H), 5.53–5.41 (m, 1H), 5.41–5.35 (m, 1H), 5.22–5.06 (m, 2H), 4.96–4.92 (m, 1H), 4.45–4.40 (m, 1H), 4.33–4.28 (m, 1H), 3.26–3.20 (m, 1H), 3.10 (2.74) (s, 3H), 2.95–2.88 (m, 1H), 2.74 (2.33) (s, 3H), 2.17–2.09 (m, 1H), 1.96–1.85 (m, 1H), 1.45 (1.32) (s, 9H), 1.11–0.90 (m, 15H). ^13^C NMR (125 MHz, CDCl_3_) δ 172.2, 171.4, 170.9, 170.5, 170.0, 168.8, 156.5, 156.2, 137.7, 137.1, 136.6, 136.6, 129.6, 129.1, 128.9, 128.7, 128.6, 128.3, 128.3, 128.2, 128.0, 127.9, 127.0, 126.7, 82.1, 81.7, 66.9, 62.0, 58.2, 57.7, 56.8, 55.5, 50.0, 47.2, 34.9, 33.3, 31.7, 31.4, 31.4, 30.8, 30.7, 29.3, 28.9, 28.0, 27.8, 19.5, 19.3, 19.0, 18.8, 17.9, 17.6, 17.5, 17.5, 14.3, 14.2. HR-ESIMS *m/z* for C_36_H_53_N_4_O_7_^+^ [M + H]^+^: calculated 653.3909, found 653.3908.

#### 3.2.5. Preparation of Hexapeptide **31**

To a solution of tetrapeptide **30** (620.0 mg, 0.95 mmol) in MeOH (30 mL), Pd/C was added under N_2_ atmosphere. The reaction vessel was sealed and flashed with H_2_ for three times. The reaction mixture was then vigirously stirred overnight under a H_2_ atmosphere. Catalyst was removed by filtration. The filtrate was concentrated in *vacuo* to give the corresponding free amine, which was pure enough and used directly in next step of reaction. To a solution of dipeptide **22** (220.0 mg, 0.43 mmol) in DCM (15 mL) was added HATU (323.0 mg, 0.85 mmol), followed by addition of DIPEA (0.4 mL, 2.13 mmol) and HOAt (116.0 mg, 0.85 mmol) at 0 °C. 0.5 h later, a solution of the above free amine in DCM (5 mL) was added at 0 °C. The reaction mixture was allowed to warm to room temperature and stirred overnight, then quenched by addition of saturated NH_4_Cl (40 mL). Layers were seperated, the aqueous phase was extracted with DCM (3 × 80 mL). The combined organic layers were washed with saturated NaHCO_3_ solution (3 × 50 mL) and brine (30 mL), dried over anhydrous Na_2_SO_4_ and concentrated *in vacuo*. The residue was purified by flash chromatography (hexane/ethyl acetate, 1:1) to afford **31** (221.0 mg, 61%). [α]_D_^25^ = +8.3 (*c* 0.8, CHCl_3_); ^1^H NMR (300 MHz, CDCl_3_) δ 7.43–7.01 (m, 11H), 6.64 (6.46) (d, *J* = 8.8 Hz, 1H), 5.49–5.34 (m, 1H), 5.33–5.17 (m, 1H), 5.17–4.95 (m, 2H), 4.85–4.80 (m, 1H), 4.78–4.43 (m, 2H), 4.43–4.18 (m, 2H), 3.27 (dd, *J* = 14.6, 5.6 Hz, 1 H), 3.12 (s, 1H), 3.10–3.01 (m, 3H), 2.97–2.93 (m, 3H), 2.90–2.83 (m, 3H), 2.80–2.77 (m, 2H), 2.31 (s, 2H), 2.26–2.09 (m, 1H), 2.01–1.85 (m, 1H), 1.80 (s, 1H), 1.41 (1.40) (s, 9H), 1.30–0.70 (m, 20 H), 0.46–0.27 (m, 1H). ^13^C NMR (75 MHz, CDCl_3_) δ 172.4, 172.3, 171.8, 171.4, 170.7, 170.4, 169.8, 168.8, 137.6, 137.0, 136.3, 129.6, 128.9, 128.9, 128.6, 128.6, 128.5, 128.1, 127.7, 126.9, 126.7, 81.7, 81.7, 67.8, 67.7, 62.1, 59.3, 58.0, 57.6, 54.3, 53.3, 52.4, 50.0, 47.7, 33.2, 32.1, 32.0, 31.5, 31.1, 31.0, 30.9, 30.8, 30.7, 30.4, 29.3, 28.9, 27.9, 19.4, 18.9, 18.5, 18.2, 18.0, 17.4, 17.2, 14.5, 14.3, 14.1, 13.6. HR-ESIMS *m/z* for C_45_H_68_N_6_NaO_10_^+^ [M + Na]^+^: calculated 875.4889, found 875.4891.

#### 3.2.6. Preparation of **32c** and **32d**

To a solution of hexapeptide **31** (40.0 mg, 0.05 mmol) in MeOH (20 mL), was added Pd/C (10% on charcoal) under N_2_ atmosphere. The reaction vessel was sealed and flashed with H_2_ for three times. The reaction mixture was then vigirously stirred overnight under H_2_ atmosphere. Catalyst was removed by filtration. The filtrate was concentrated in *vacuo* to give the corresponding free amine, which was pure enough and used directly in next step of reaction. To a solution of *S*-**3** (23.0 mg, 0.14 mmol) in DCM (2 mL) was added HATU (34.0 mg, 0.09 mmol), followed by addition of DIPEA (39 μL, 0.23 mmol) and HOAt (12.0 mg, 0.09 mmol) at 0 °C. 0.5 h later, a solution of the above amine (32.0 mg, 0.04 mmol) in DCM (2 mL) was added at 0 °C. The reaction mixture was allowed to warm to room temperature and stirred overnight. The reaction was quenched with saturated NH_4_Cl (20 mL), and extracted with DCM (3 × 50 mL). The combined organic layers were washed with saturated NaHCO_3_ solution (3 × 50 mL) and brine (50 mL), dried over anhydrous Na_2_SO_4_ and concentrated *in vacuo*. The residue was purified by flash chromatography (ethyl acetate) to give compound **32c** (20.0 mg, 50%). [*α*]_D_^25^ = −12.3 (*c* 0.1, CHCl_3_); ^1^H NMR (400 MHz, CDCl_3_) δ 8.07 (d, *J* = 9.0 Hz, 0.5H), 7.32–7.28 (m, 1H), 7.26–7.14 (m, 4H), 6.96 (d, *J* = 9.5 Hz, 0.4H), 6.68 (d, *J* = 8.9 Hz, 0.4H), 6.50 (d, *J* = 8.9 Hz, 0.6H), 6.08–6.01 (m, 1H), 5.52–5.37 (m, 1H), 5.36–5.30 (m, 1H), 5.14–5.01 (m, 1H), 4.91–4.65 (m, 1H), 4.59–4.42 (m, 1H), 4.30 (dd, *J* = 8.8, 4.6 Hz, 2H), 3.31–3.18 (m, 1H), 3.17–3.08 (m, 1H), 3.07–2.82 (m, 9H), 2.80–2.74 (m, 3H), 2.51–2.37 (m, 1H), 2.37–2.29 (m, 2H), 2.28–2.17 (m, 1H), 2.17–2.07 (m, 1H), 2.02–1.84 (m, 2H), 1.43 (s, 9H), 1.39–1.28 (m, 3H), 1.19–1.07 (m, 6H), 1.05–0.97 (m, 3H), 0.91–0.76 (m, 12H). ^13^C NMR (75 MHz, CDCl_3_) δ 172.5, 172.4, 172.3, 171.9, 171.8, 171.4, 170.7, 170.4, 169.9, 168.8, 156.8, 137.6, 137.0, 136.3, 129.6, 128.9, 128.9, 128.6, 128.5, 128.1, 127.7, 126.9, 126.7, 81.7, 68.5, 68.2, 68.0, 67.8, 62.9, 62.1, 60.3, 59.3, 58.9, 58.2, 58.0, 57.9, 57.6, 56.9, 54.3, 53.3, 52.4, 50.9, 50.0, 47.7, 47.5, 43.2, 34.7, 33.2, 32.1, 32.0, 31.5, 31.1, 31.0, 30.7, 30.4, 29.3, 28.9, 27.9, 22.4, 19.4, 19.4, 18.9, 18.5, 18.2, 18.0, 17.4, 17.2, 15.0, 14.5, 14.3, 14.1, 13.6. HR-ESIMS *m/z* for C_42_H_68_Cl_2_N_6_NaO_9_^+^ [M + Na]^+^: calculated 893.4317, found 893.4318.

Compound **32d** was prepared in 52% yield. Analytical data: [*α*]_D_^25^ = −28.2 (*c* 0.3, CHCl_3_); ^1^H NMR (300 MHz, CDCl_3_) δ 8.09 (d, *J* = 9.0 Hz, 0.5H), 7.43–7.13 (m, 5H), 7.00 (d, *J* = 9.5 Hz, 0.5H), 6.70 (d, *J* = 8.9 Hz, 0.5H), 6.52 (d, *J* = 8.9 Hz, 0.5H), 6.15–5.91 (m, 1H), 5.58–5.38 (m, 1H), 5.38–5.23 (m, 1H), 5.16–5.01 (m, 1H), 4.93–4.65 (m, 1H), 4.54 (ddd, *J* = 14.3, 8.9, 5.8 Hz, 1H), 4.32 (dd, *J* = 8.8, 4.6 Hz, 2H), 3.26 (dt, *J* = 16.8, 7.2 Hz, 1H), 3.17–3.07 (m, 3H), 3.07–2.90 (m, 3H), 2.90 (2.88) (s, 3H), 2.86–2.76 (m, 3H), 2.71 (dt, *J* = 16.7, 6.2 Hz, 1H), 2.55–2.42 (m, 1H), 2.32 (s, 1H), 2.17 (ddd, *J* = 14.0, 12.4, 6.6 Hz, 1H), 2.03–1.87 (m, 1H), 1.46 (s, 9H), 1.39–1.35 (m, 3H), 1.26–1.22 (m, 6H), 1.15–1.11 (m, 3H), 1.04 (t, *J* = 6.5 Hz, 3H), 0.93–0.77 (m, 9H), 0.38 (d, *J* = 6.9 Hz, 1H). ^13^C NMR (75 MHz, CDCl_3_) δ 172.5, 172.4, 172.3, 172.0, 171.9, 171.4, 171.2, 171.0, 170.7, 170.6, 170.4, 169.9, 169.8, 168.9, 137.6, 137.0, 129.6, 129.0, 128.9, 128.7, 127.0, 126.7, 84.6, 81.7, 68.6, 62.0, 58.0, 57.9, 57.6, 57.3, 57.0, 55.7, 54.3, 52.6, 52.4, 51.7, 50.0, 48.8, 47.8, 45.6, 40.3, 35.6, 35.6, 34.7, 33.3, 31.5, 31.2, 31.0, 30.9, 30.7, 30.7, 30.6, 30.5, 29.6, 29.3, 28.9, 27.9, 19.4, 19.4, 18.9, 18.7, 18.5, 18.0, 17.4, 17.3, 15.2, 14.6, 14.3, 14.1, 13.7. HR-ESIMS *m/z* for C_42_H_68_Cl_2_N_6_NaO_9_^+^ [M + Na]^+^: calculated 893.4317, found 893.4316.

#### 3.2.7. Completion of the Synthesis of Itralamide B **1c** and **1d**

To a solution of compound **32c** (15.0 mg, 0.02 mmol) in DCM (1.0 mL), BF_3_^.^Et_2_O (21 μL, 0.17 mmol) was added dropwise at 0 °C. The reaction solution was then allowed to warm to room temperature and stirred for 0.5~1.0 h (monitored by TLC). The reaction was quenched by addition of saturated NH_4_Cl (2 mL) and diluted with DCM (60 mL). The organic phase was washed with saturated NH_4_Cl (3 × 20 mL) and brine (20 mL), dried over anhydrous Na_2_SO_4_ and concentrated in *vacuo* to give crude hydroxy acid, which was dried under high vacuum for 4 h. To a solution of DMAP (21.0 mg, 0.17 mmol) and MNBA (30.0 mg, 0.08 mmol), a solution of above hydroxy acid in toluene (5 mL) was slowly added at 0 °C. After the reaction mixture was warmed to room temperature, it was gradually heated to 60 °C and stirred for two days. The reaction mixture was diluted with ethyl acetate (100 mL) and washed successively with saturated NH_4_Cl (3 × 20 mL) and brine (2 × 20mL), dried over anhydrous Na_2_SO_4_ and concentrated *in vacuo*. The residue was purified by flash chromatography (ethyl acetate) to afford itralamide B **1c** (2.7 mg, 20%). [α]_D_^25^ = −10.8 (*c* 0.1, CHCl_3_); ^1^H NMR (600 MHz, CDCl_3_) δ 8.45 (d, *J* = 7.6 Hz, 1H), 7.33–7.28 (m, 2H), 7.19 (d, *J* = 7.0 Hz, 3H), 5.98 (d, *J* = 3.0 Hz, 1H), 5.80 (d, *J* = 3.3 Hz, 1H), 5.64 (dd, *J* = 6.7, 3.1 Hz, 1H), 5.34 (dd, *J* = 11.4, 3.7 Hz, 1H), 5.12 (q, *J* = 6.9 Hz, 1H), 4.98 (q, *J* = 6.7 Hz, 1H), 4.67 (t, *J* = 10.1 Hz, 1H), 4.63 (dd, *J* = 7.5, 3.8 Hz, 1H), 3.36 (s, 3H), 3.23–3.10 (m, 2H), 3.08 (s, 3H), 3.05 (s, 3H), 2.99 (s, 3H), 2.91 (dd, *J* = 14.3, 3.4 Hz, 2H), 2.85 (d, *J* = 8.9 Hz, 1H), 2.81–2.74 (m, 2H), 2.70 (dd, *J* = 16.6, 5.1 Hz, 2H), 2.47 (dd, *J* = 16.7, 7.6 Hz, 1H), 2.32–2.27 (m, 1H), 2.27–2.21 (m, 1H), 2.02 (s, 1H), 1.36 (d, *J* = 6.7 Hz, 3H), 1.20 (d, *J* = 6.6 Hz, 3H), 1.05 (d, *J* = 6.9 Hz, 3H), 0.97 (d, *J* = 6.7 Hz, 3H), 0.95 (d, *J* = 6.8 Hz, 3H), 0.90 (s, 1H), 0.88 (d, *J* = 6.8 Hz, 3H), 0.41 (d, *J* = 6.8 Hz, 2H). ^13^C NMR (150 MHz, CDCl_3_) δ 172.4, 172.2, 171.4, 170.2, 169.5, 168.8, 137.2, 132.1, 132.0, 131.9, 129.7, 128.9, 128.5, 128.4, 127.0, 78.0, 69.8, 61.2, 57.6, 55.5, 53.5, 52.7, 46.6, 40.6, 35.7, 35.7, 34.1, 32.5, 31.9, 31.4, 30.7, 29.8, 29.7, 29.6, 29.4, 28.8, 22.7, 19.6, 18.8, 18.7, 18.0, 17.6, 15.4. HR-ESIMS *m/z* for C_38_H_58_Cl_2_N_6_NaO_8_^+^ [M + Na]^+^: calculated 819.3585, found 819.3587.

Compound itralamide B **1d** was prepared in 21% yield. Analytical data: [α]_D_^25^ = −8.3 (*c* 0.1, CHCl_3_); ^1^H NMR (600 MHz, CDCl_3_) δ 8.46 (d, *J* = 7.5 Hz, 1H), 7.32 (dd, *J* = 9.6, 5.4 Hz, 2H), 7.20 (d, *J* = 7.1 Hz, 3H), 6.00 (d, *J* = 2.9 Hz, 1H), 5.82 (d, *J* = 3.1 Hz, 1H), 5.66 (dd, *J* = 6.7, 3.2 Hz, 1H), 5.41–5.29 (m, 2H), 5.14 (q, *J* = 6.9 Hz, 1H), 5.00 (q, *J* = 6.8 Hz, 1H), 4.69 (t, *J* = 10.1 Hz, 1H), 4.64 (dd, *J* = 7.6, 3.8 Hz, 1H), 3.38 (s, 3H), 3.19 (dd, *J* = 14.0, 11.5 Hz, 1H), 3.10 (s, 3H), 3.07 (s, 3H), 3.01 (s, 3H), 2.98–2.94 (m, 2H), 2.94–2.90 (m, 1H), 2.80 (d, *J* = 18.3 Hz, 2H), 2.72 (dd, *J* = 16.6, 5.1 Hz, 1H), 2.49 (dd, *J* = 16.7, 7.6 Hz, 1H), 2.38 (d, *J* = 6.4 Hz, 1H), 2.34–2.30 (m, 1H), 2.25 (dd, *J* = 9.7, 5.7 Hz, 1H), 2.03 (s, 1H), 1.38 (d, *J* = 6.7 Hz, 3H), 1.22 (d, *J* = 6.6 Hz, 3H), 1.07 (d, *J* = 7.0 Hz, 3H), 0.99 (d, *J* = 6.7 Hz, 3H), 0.97 (d, *J* = 6.9 Hz, 3H), 0.92 (s, 1H), 0.90 (d, *J* = 6.7 Hz, 3H), 0.43 (d, *J* = 6.8 Hz, 2H). ^13^C NMR (150 MHz, CDCl_3_) δ 172.8, 172.4, 172.2, 171.4, 170.2, 169.5, 168.8, 137.2, 129.7, 128.9, 127.0, 78.0, 69.8, 61.2, 57.6, 55.5, 53.5, 52.7, 46.6, 40.6, 35.9, 35.7, 34.2, 32.5, 31.9, 31.9, 31.4, 30.7, 29.8, 29.7, 29.3, 28.8, 22.7, 19.6, 18.8, 18.7, 18.0, 17.6, 15.4, 14.2, 14.1, 13.4. HR-ESIMS *m/z* for C_38_H_58_Cl_2_N_6_NaO_8_^+^ [M + Na]^+^: calculated 819.3585, found 819.3589.

#### 3.2.8. Analytical Data of Itralamide B **1a** and **1b** [26]

Itralamide B **1a**: [α]_D_^25^ = −50.9 (*c* 0.1, CHCl_3_); ^1^H NMR (400 MHz, CDCl_3_) δ 7.45–7.12 (m, 5H), 6.90 (d, *J* = 9.6 Hz, 1H), 6.47 (d, *J* = 8.0 Hz, 1H), 5.99 (d, *J* = 2.9 Hz, 1H), 5.75–5.65 (m, 2H), 5.53–5.43 (m, 1H), 5.08 (q, *J* = 6.8 Hz, 1H), 4.98 (dd, *J* = 7.9, 4.2 Hz, 1H), 4.71 (dd, *J* = 9.4, 4.1 Hz, 1H), 4.62 (q, *J* = 7.6 Hz, 1H), 3.66 (dd, *J* = 15.5, 4.9 Hz, 1H), 3.33 (d, *J* = 23.6 Hz, 3H), 3.19 (d, *J* = 7.1 Hz, 3H), 3.17–3.07 (m, 3H), 3.07–2.94 (m, 3H), 2.87 (dd, *J* = 15.6, 12.1 Hz, 1H), 2.80 (d, *J* = 4.0 Hz, 1H), 2.76–2.65 (m, 1H), 2.46 (dd, *J* = 16.5, 7.5 Hz, 1H), 2.28–2.21 (m, 1H), 2.06–1.99 (m, 1H), 1.29 (d, *J* = 2.0 Hz, 3H), 1.26 (d, *J* = 2.8 Hz, 3H), 1.20 (dd, *J* = 6.6, 3.5 Hz, 3H), 1.08–1.01 (m, 6H), 0.92 (d, *J* = 7.2 Hz, 6H), 0.80 (d, *J* = 6.8 Hz, 3H). ^13^C NMR (100 MHz, CDCl_3_) δ 174.9, 172.8, 172.5, 170.6, 170.2, 169.9, 169.5, 137.4, 128.5, 128.3, 126.5, 78.2, 69.6, 56.9, 56.8, 54.7, 54.7, 54.0, 51.4, 40.6, 35.8, 33.9, 33.8, 32.2, 31.8, 31.1, 31.0, 30.5, 19.9, 19.6, 17.8, 17.1, 15.6, 15.4, 14.1, 13.8. HR-ESIMS *m/z* for C_38_H_58_Cl_2_N_6_NaO_8_^+^ [M + Na]^+^: calculated 819.3585, found 819.3587.

Itralamide B **1b**: [α]_D_^25^ = −42.3 (*c* 0.2, CHCl_3_); ^1^H NMR (400 MHz, CDCl_3_) δ 7.24–7.16 (m, 5H), 6.89 (d, *J* = 9.1 Hz, 1H), 6.48 (d, *J* = 7.8 Hz, 1H), 6.04 (d, *J* = 2.9 Hz, 1H), 5.78–5.63 (m, 1H), 5.49 (dd, *J* = 6.6, 3.2 Hz, 1H), 5.45–5.35 (m, 1H), 5.12–5.02 (m, 1H), 4.98 (dd, *J* = 7.8, 4.2 Hz, 1H), 4.71 (dd, *J* = 9.3, 4.0 Hz, 1H), 4.67–4.55 (m, 1H), 3.66 (dd, *J* = 15.3, 5.1 Hz, 1H), 3.28 (d, *J* = 63.9 Hz, 3H), 3.18 (s, 3H), 3.13 (d, *J* = 23.1 Hz, 3H), 3.01 (d, *J* = 11.3 Hz, 3H), 2.93 (d, *J* = 15.7 Hz, 1H), 2.90–2.78 (m, 1H), 2.78–2.67 (m, 1H), 2.43 (dd, *J* = 16.7, 6.0 Hz, 1H), 2.25 (dd, *J* = 11.9, 5.9 Hz, 1H), 2.04 (d, *J* = 9.5 Hz, 1H), 1.38 (d, *J* = 7.2 Hz, 3H), 1.30 (d, *J* = 6.7 Hz, 3H), 1.20 (d, *J* = 6.6 Hz, 3H), 1.06 (d, *J* = 6.8 Hz, 6H), 0.92 (d, *J* = 7.0 Hz, 6H), 0.80 (d, *J* = 6.8 Hz, 3H). ^13^C NMR (100 MHz, CDCl_3_) δ 174.9, 172.8, 172.6, 170.7, 170.2, 169.8, 169.5, 137.4, 128.6, 128.3, 126.5, 78.0, 69.6, 56.9, 56.8, 54.8, 54.2, 54.0, 51.5, 40.4, 36.0, 34.0, 33.8, 32.2, 31.8, 31.1, 31.0, 30.6, 19.9, 19.6, 17.8, 17.1, 17.1, 15.6, 15.0, 13.8. HR-ESIMS *m/z* for C_38_H_58_Cl_2_N_6_NaO_8_^+^ [M + Na]^+^: calculated 819.3585, found 819.3589.

### 3.3. Biological Test

Cell proliferation assay: Neuroblastoma cell line SH-SY5Y, cervical adenocarcinoma cell line HeLa, and hepatocellular carcinoma cell lines Hep3B and PLC were obtained from American Type Culture Collection (Manassas, VA, USA), and cultured in DMEM containing supplements (10% FBS, penicillin/streptomycin and l-glutamine). Cells were seeded into 96-well plates overnight and cultured with incremental concentrations of the compounds in the medium containing 1% FBS for another 72 h. The effect of the compounds was evaluated by cell proliferation assay using 3-(4,5-dimethylthiazol-2-yl)-5-(3-carboxymethoxyphenyl)-2-(4-sulfophenyl-2H-tetrazolium) (MTS) assay (Promega Co., Madison, WI, USA). According to the manufacturer’s instructions, 20 μL of CellTiter96 Aqueous solution was added into each well containing 100 μL medium and incubated at 37 °C for 4 h. The absorbance at 490 nm was measured using an ELISA plate reader (Bio-Rad microplate reader 680, Bio-Rad Laboratories, Hercules, CA, USA). IC_50_ values were calculated using GraphPad Prism software (GraphPad Prism software Inc., La Jolla, CA, USA).

## 4. Conclusions

A reliable and convergent strategy for the total synthesis of itralamide B had been developed. Four stereoisomers of itralamide B **1a**–**1d** were prepared. Comparison of spectral data for the synthetic samples **1a**–**1d** with data on itralamide B in the literature revealed significant differences, and these discrepancies led to some uncertainty concerning the structure of itralamide B. The current work proved that these data discrepancies originated somewhere other than the configuration of the valine residues. Further work is still required to determine the true structure of natural itralamide B. Furthermore, itralamide B **1a**–**1d** and compound **16** were evaluated using cell proliferation assay, which revealed that stereoisomers **1a**, **1c** and **1d** showed moderate inhibitory activity toward PLC cancer cell.

## Supplementary Files

Supplementary File 1
