# Supplementary material for: Studies toward the Total Synthesis of Itralamide B and Biological Evaluation of Its Structural Analogs
Source: Mar Drugs. 2015 Apr 13;13(4):2085–104. doi: 10.3390/md13042085 (PMC4413201; doi:10.3390/md13042085)
Supplement: Supplementary File 1 [file marinedrugs-13-02085-s001.pdf]

Supplementary Information

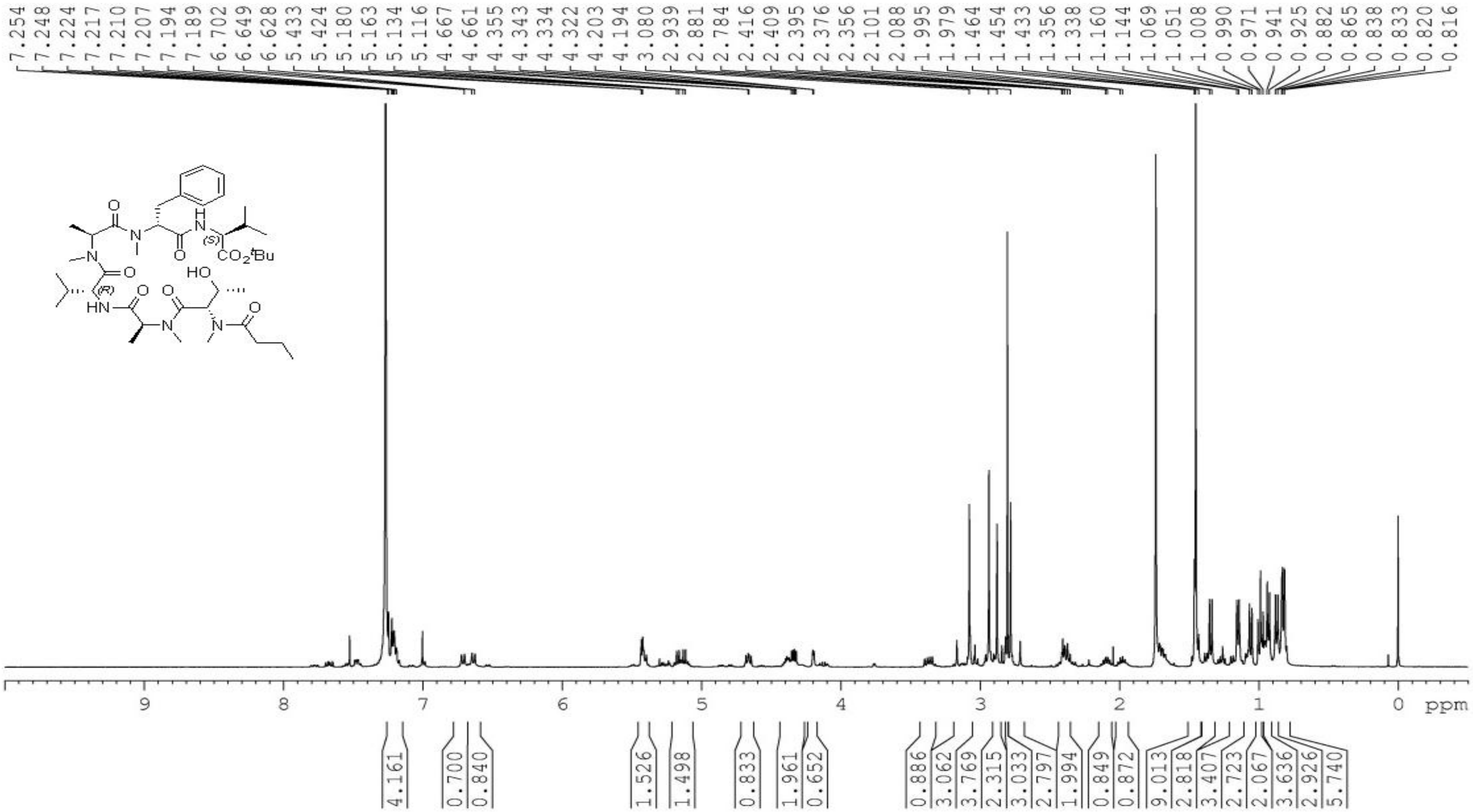

Figure S1. ^{1}H NMR of compound **19**.

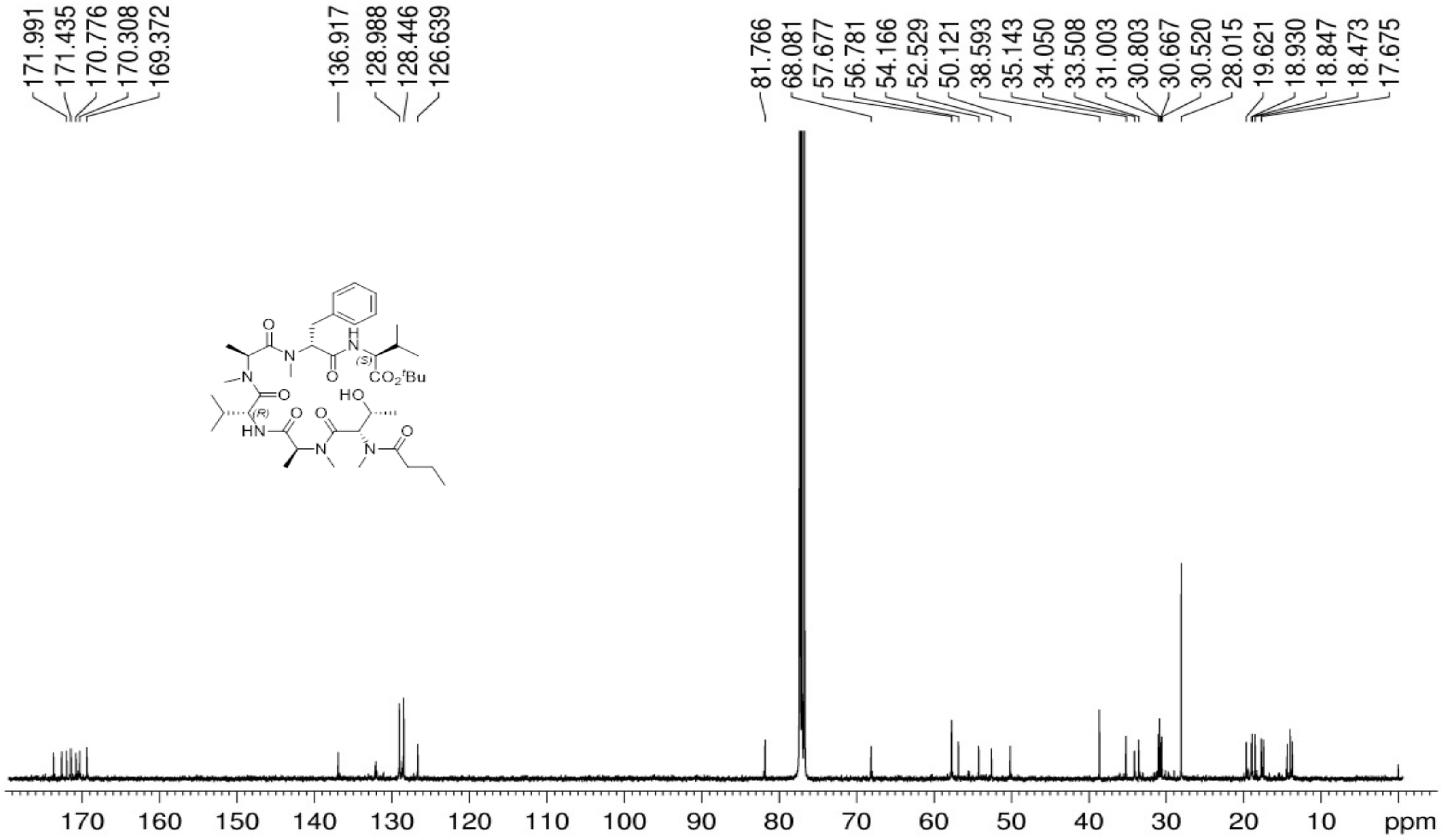

Figure S2. ^{13}C NMR of compound **19**.

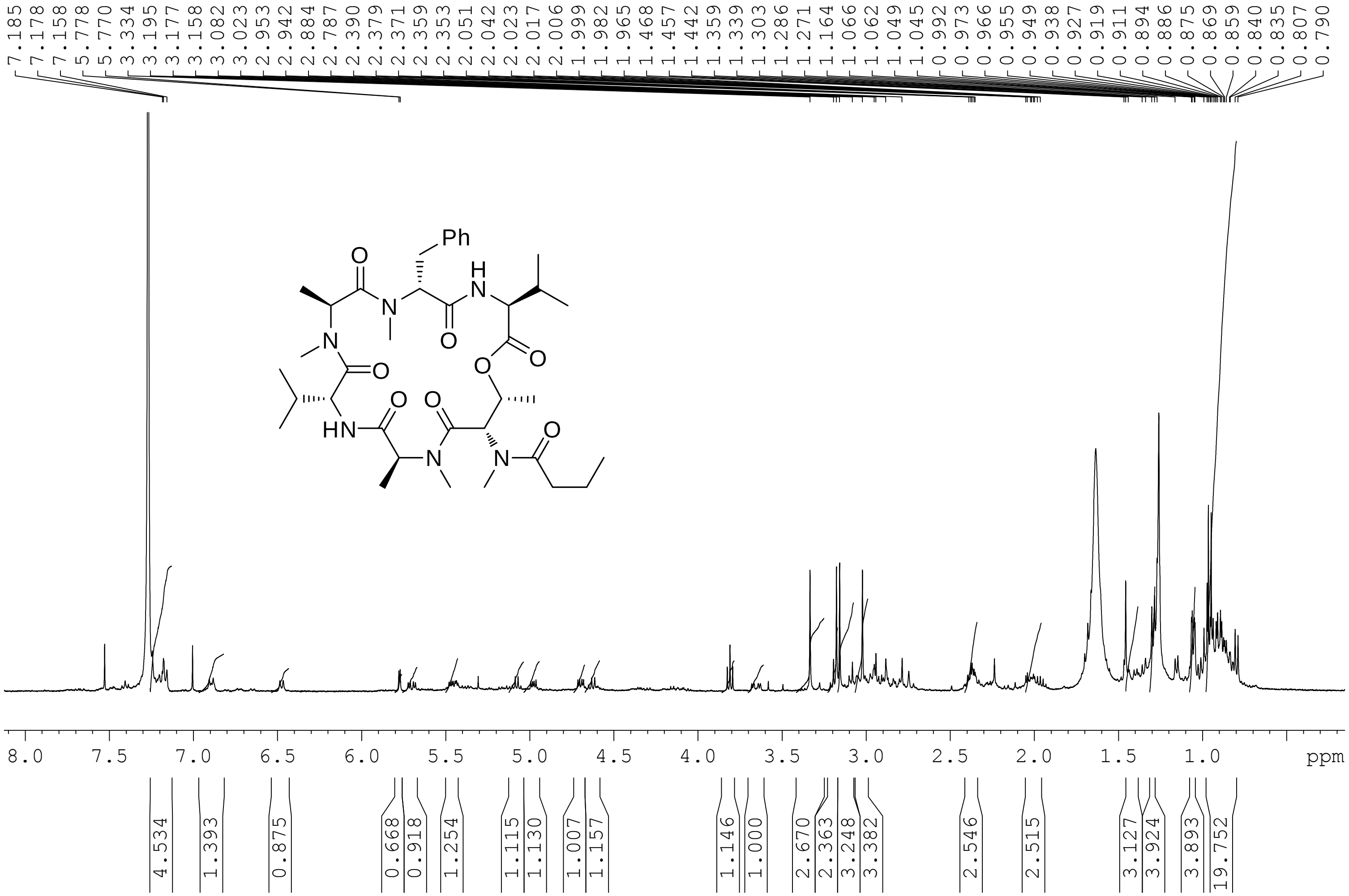

Figure S3. ^{1}H NMR of compound **16**.

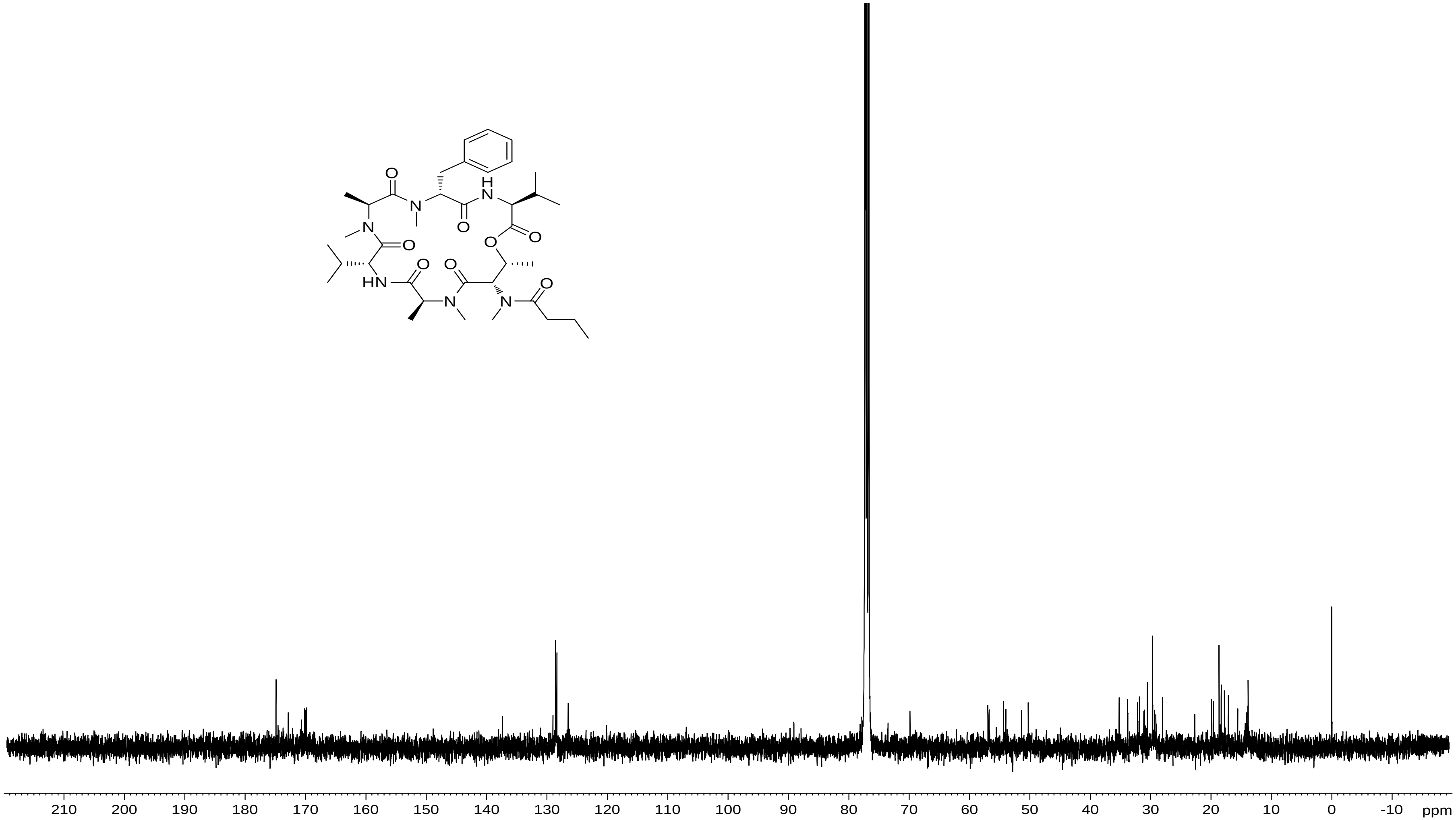

Figure S4. ^{13}C NMR of compound **16**.

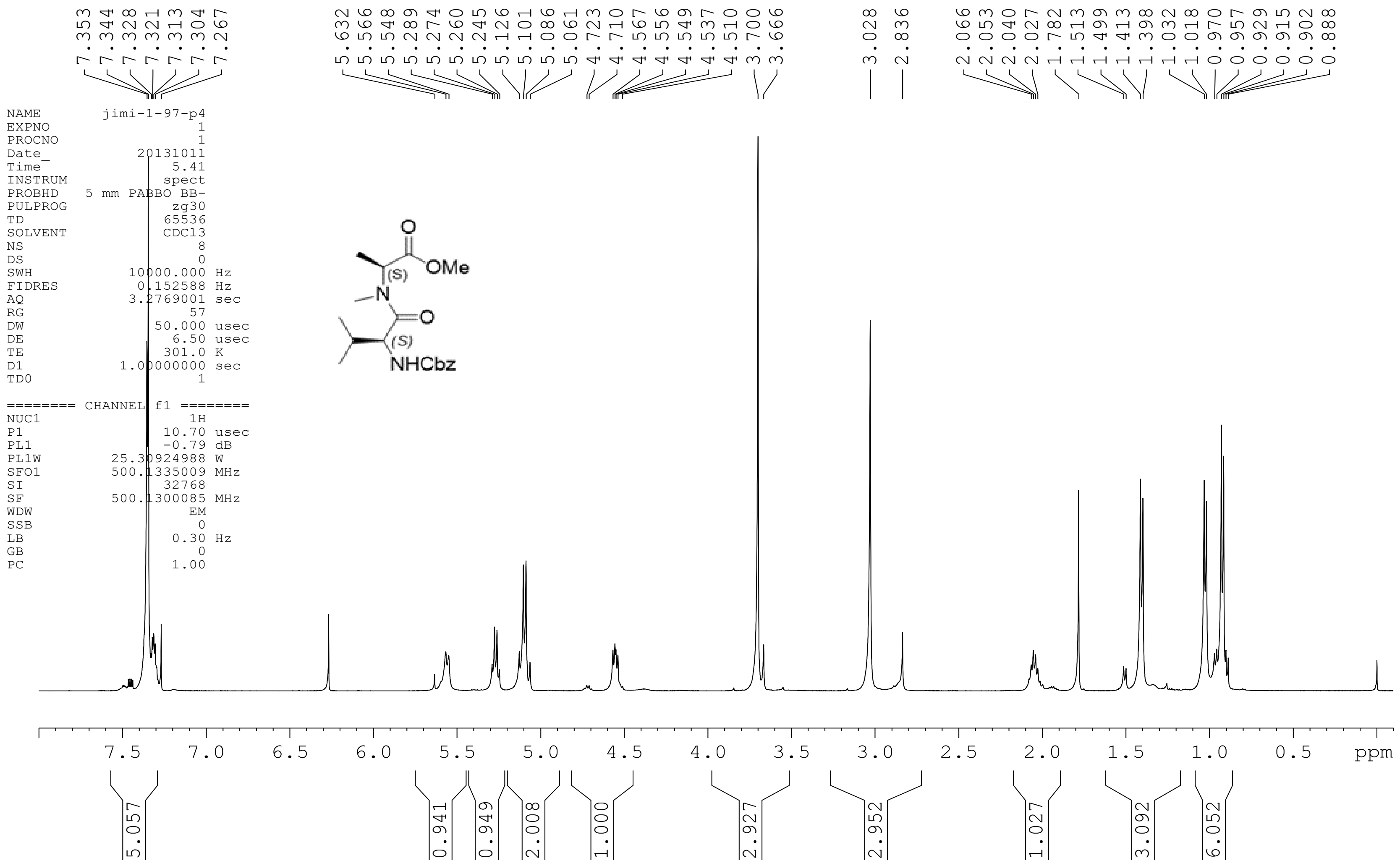

Figure S5. ^{1}H NMR of dipeptide Cbz-L-Val-N(Me)-Ala-OMe.

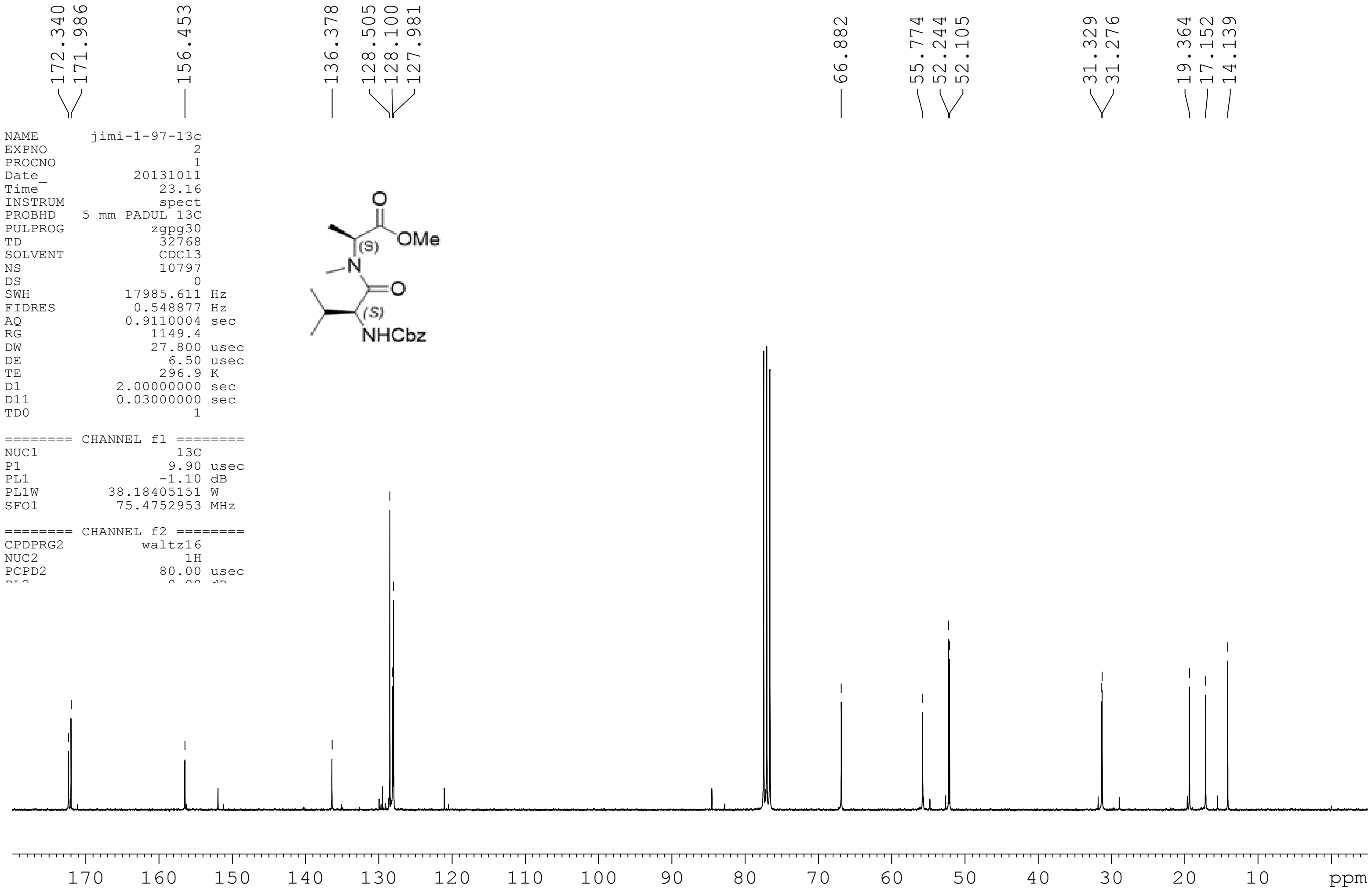

Figure S6. ^{13}C NMR of dipeptide Cbz-L-Val-N(Me)-Ala-OMe.

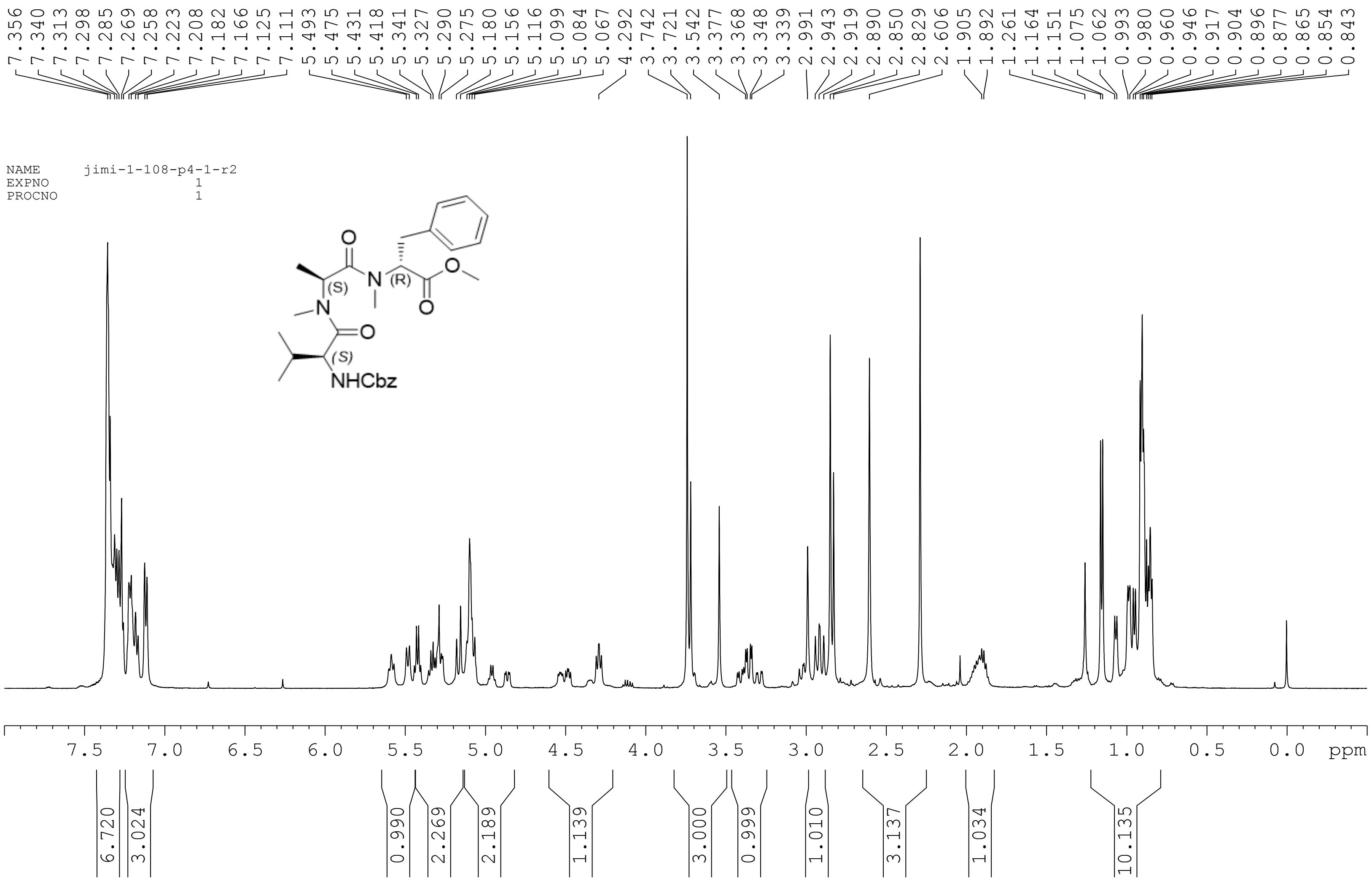

Figure S7. ^{1}H NMR of compound **28**.

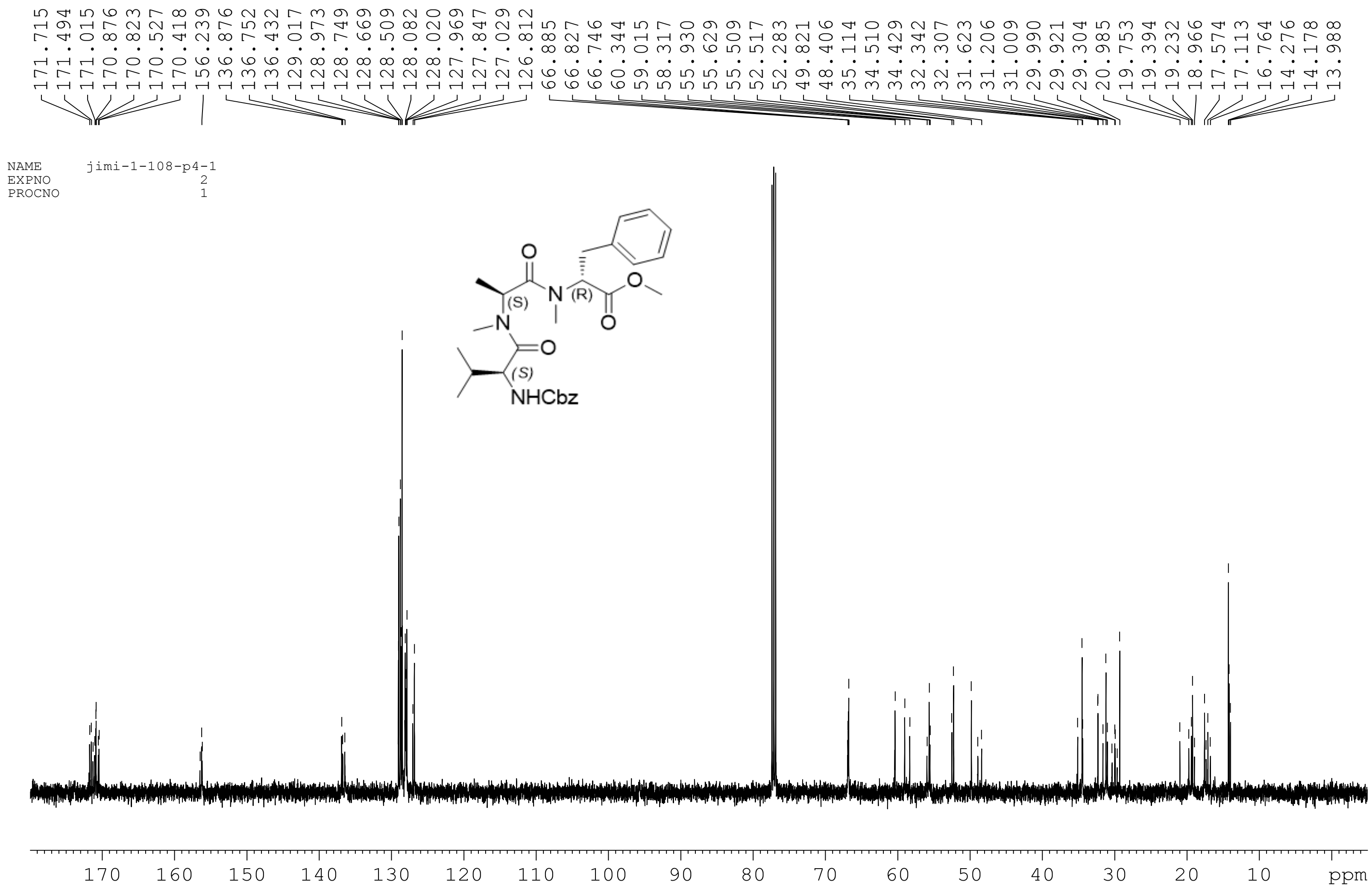

Figure S8. ^{13}C NMR of compound **28**.

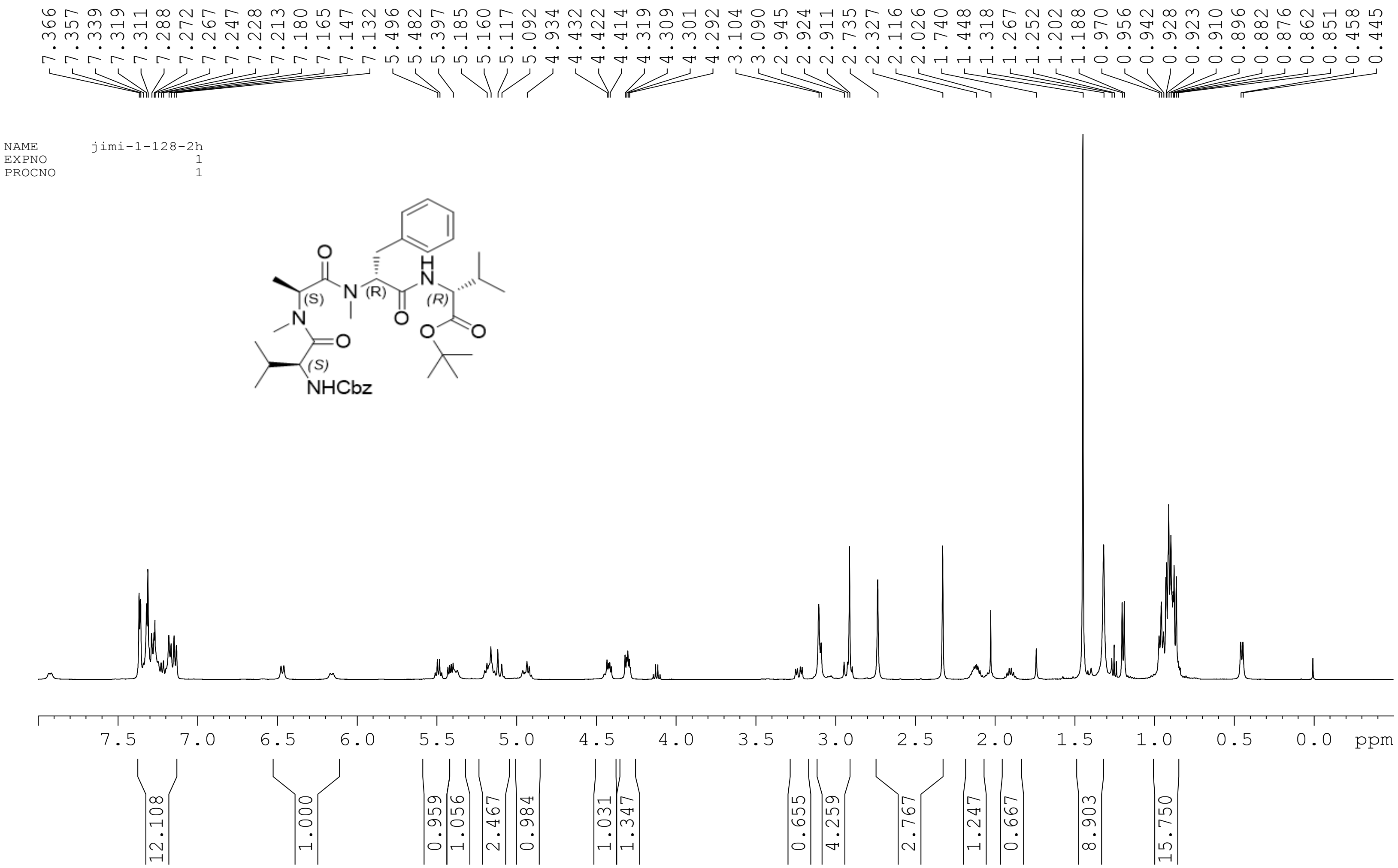

Figure S9. ^{1}H NMR of compound **30**.

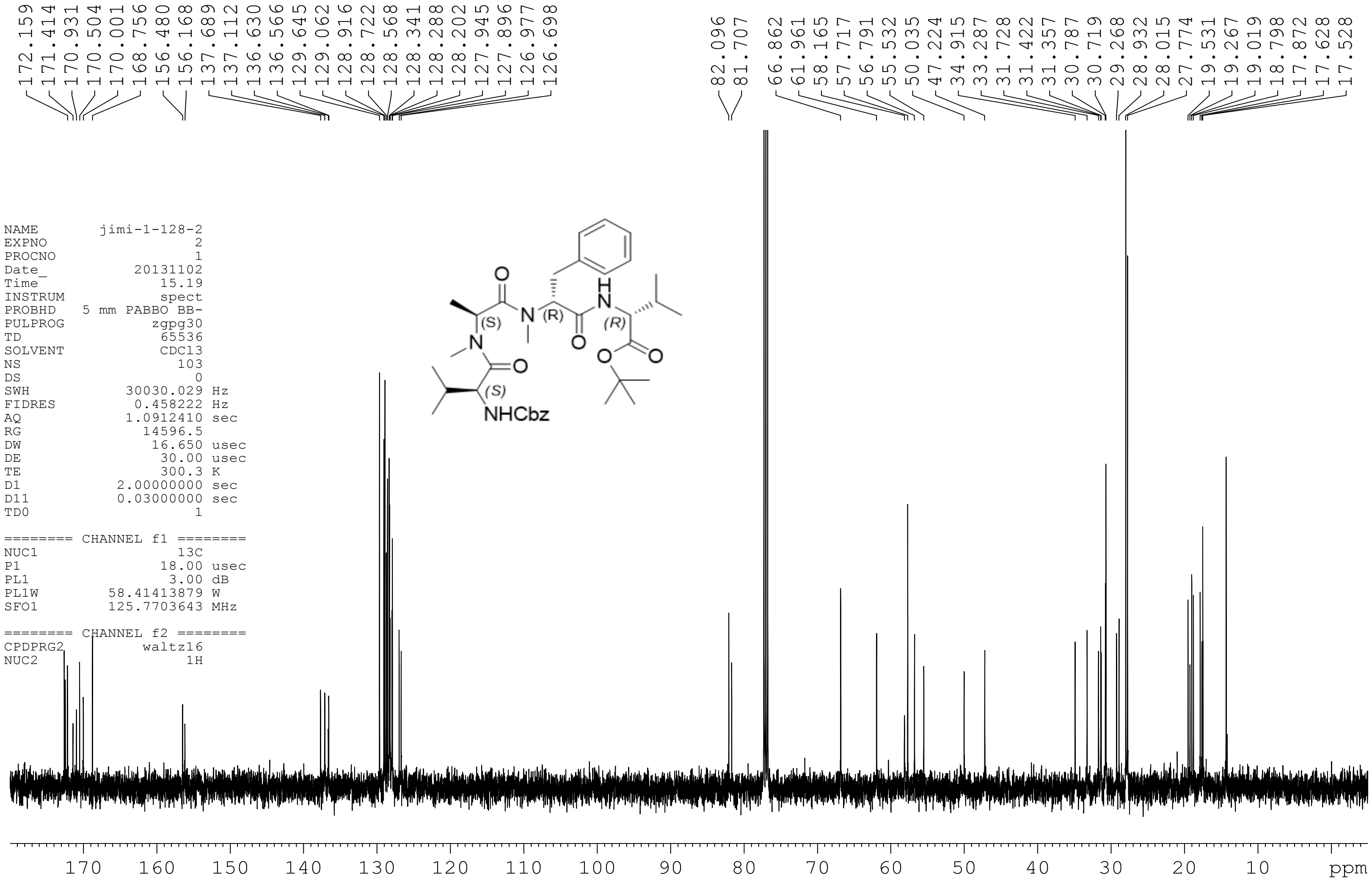

Figure S10. ^{13}C NMR of compound **30**.

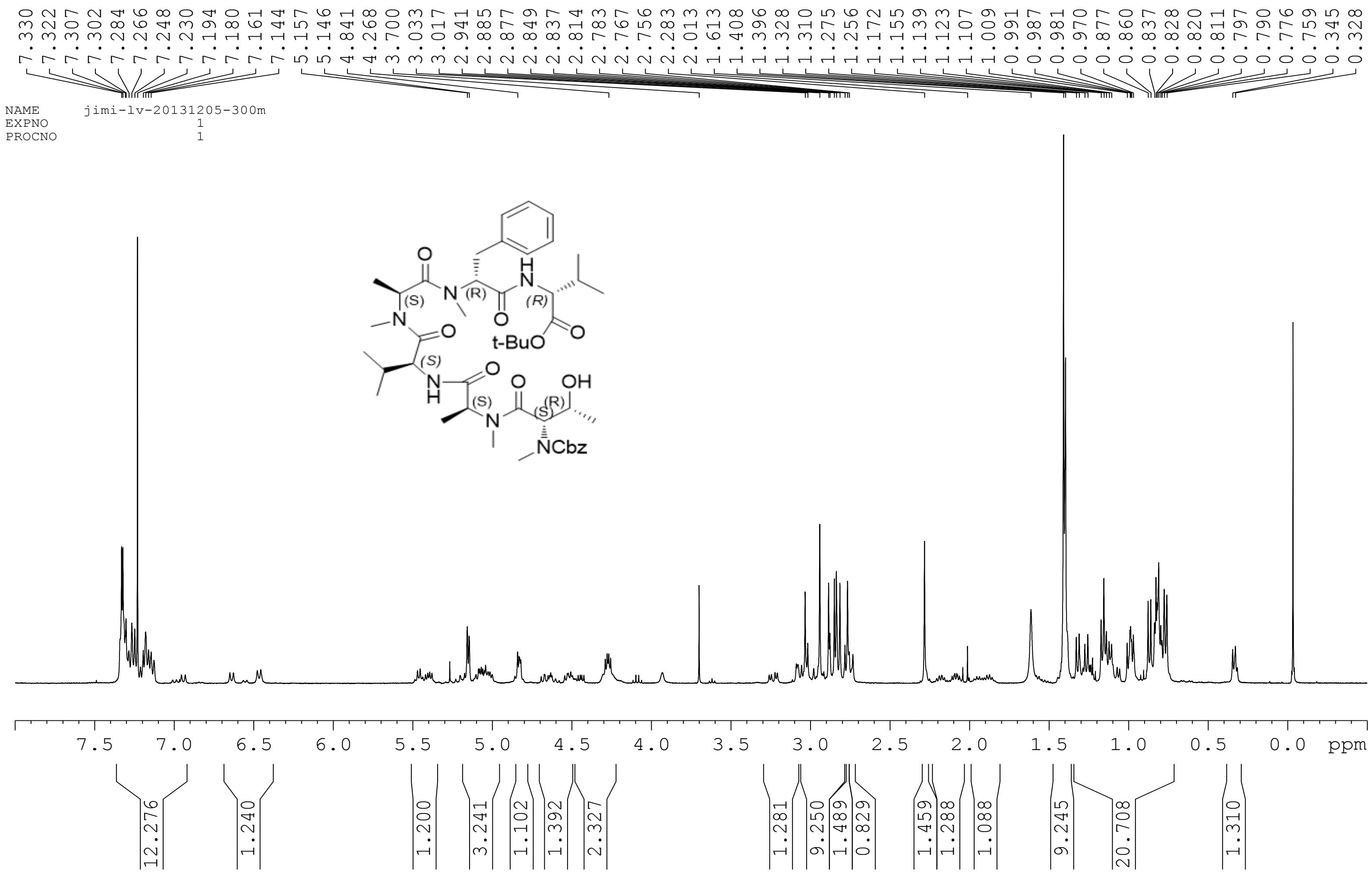

Figure S11. ^{1}H NMR of compound **31**.

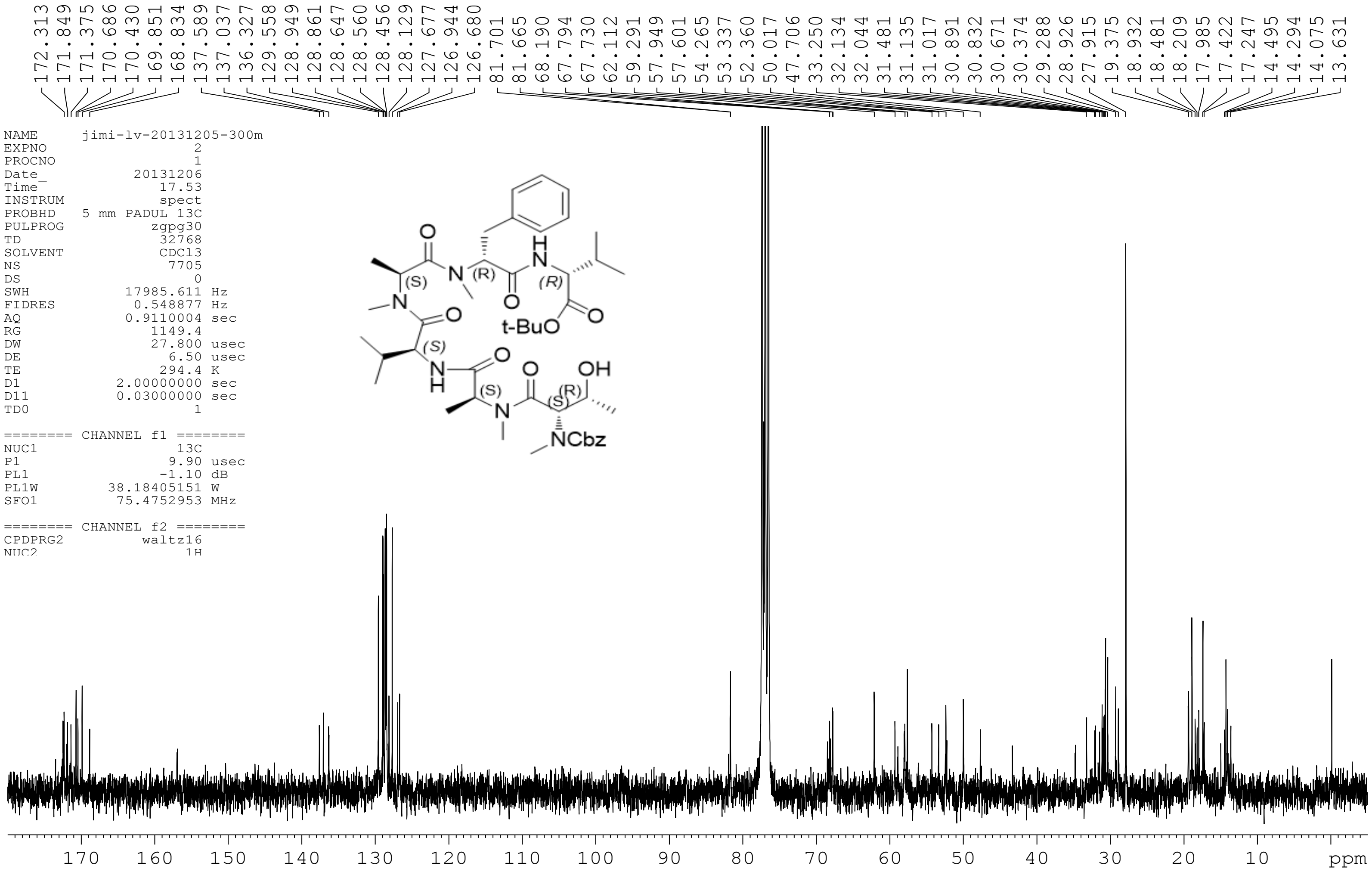

Figure S12. ^{13}C NMR of compound **31**.

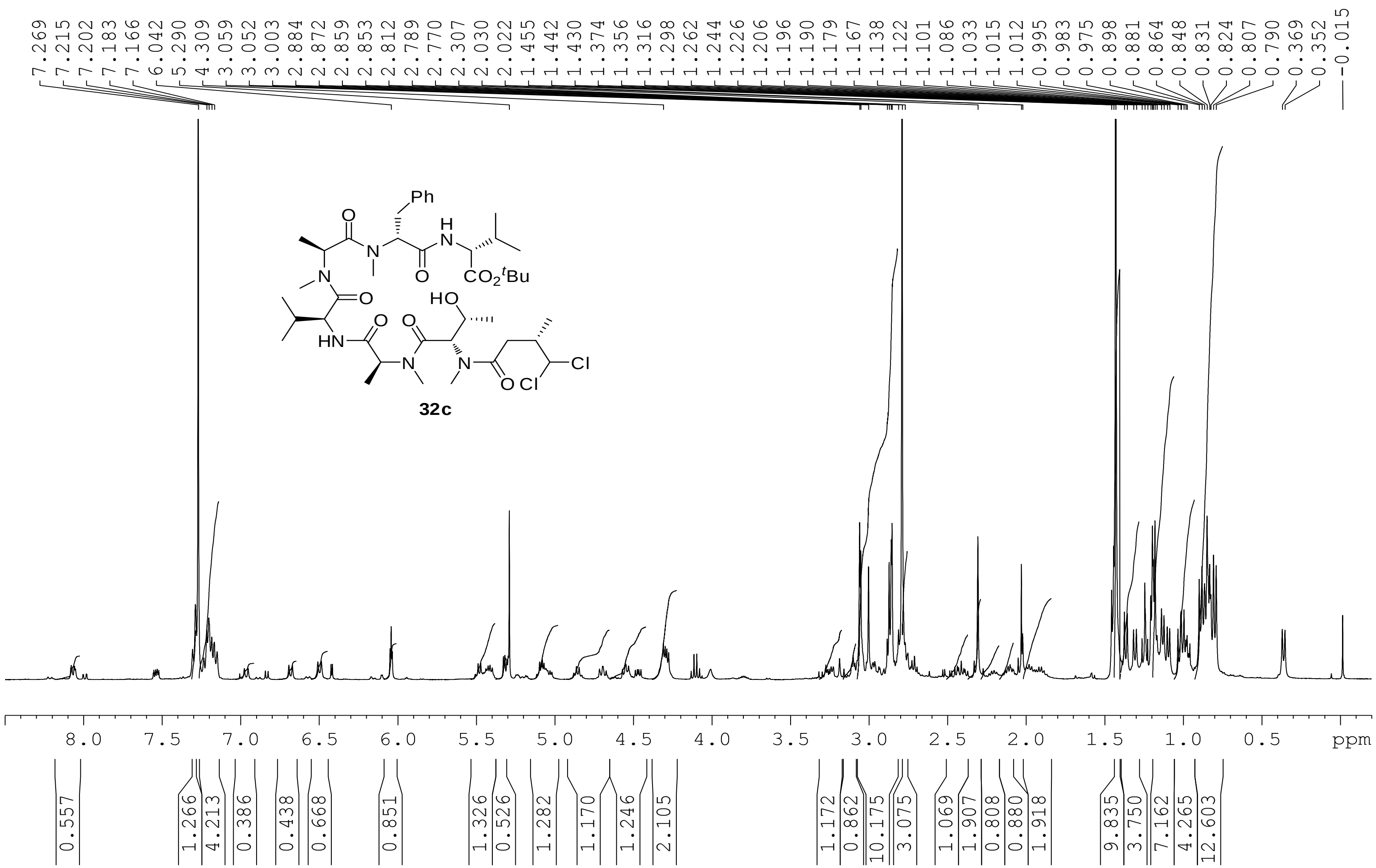

Figure S13. ^{1}H NMR of compound **32c**.

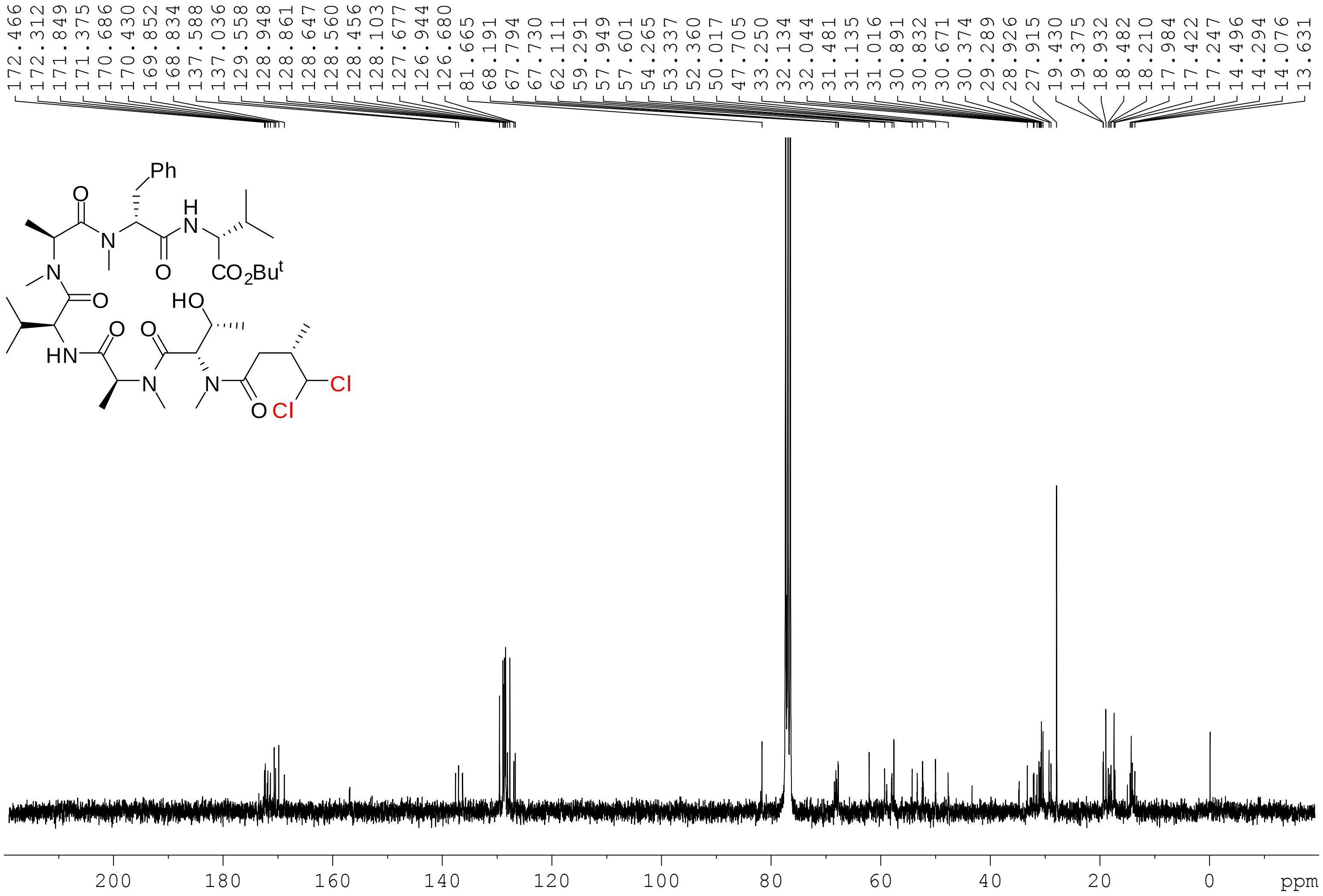

Figure S14. ^{13}C NMR of compound **32c**.

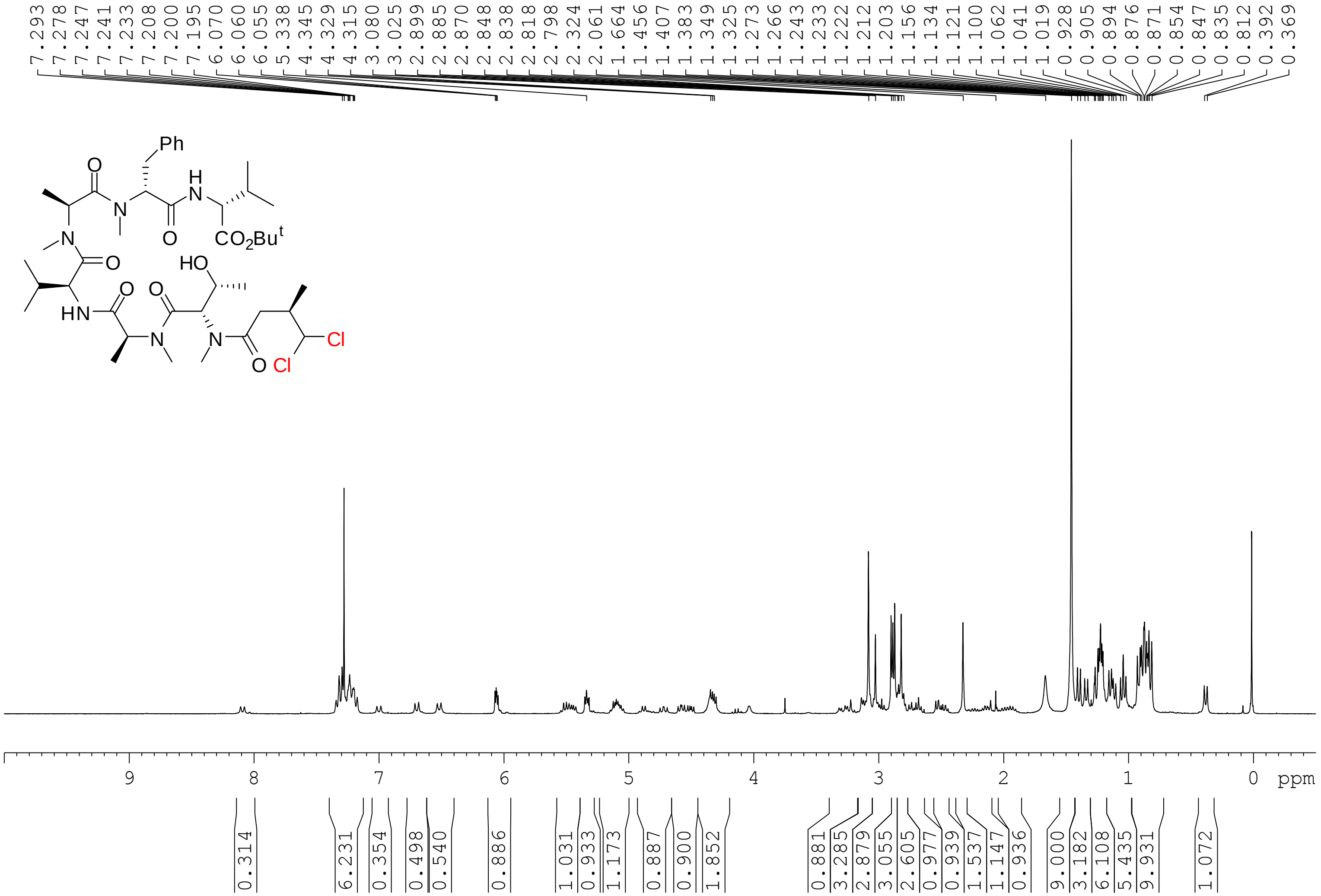

Figure S15. ^{1}H NMR of compound **32d**.

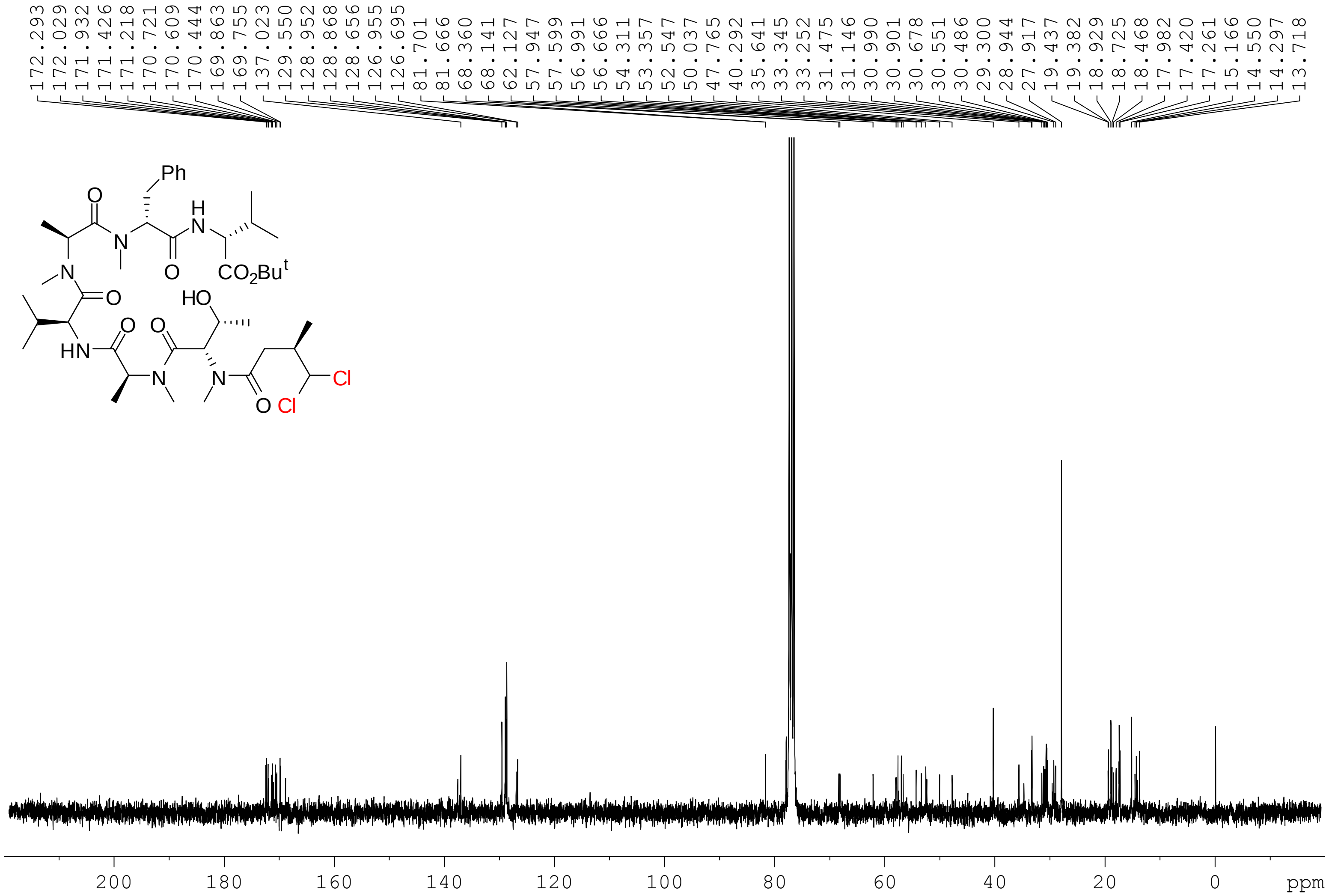

Figure S16. ^{13}C NMR of compound **32d**.

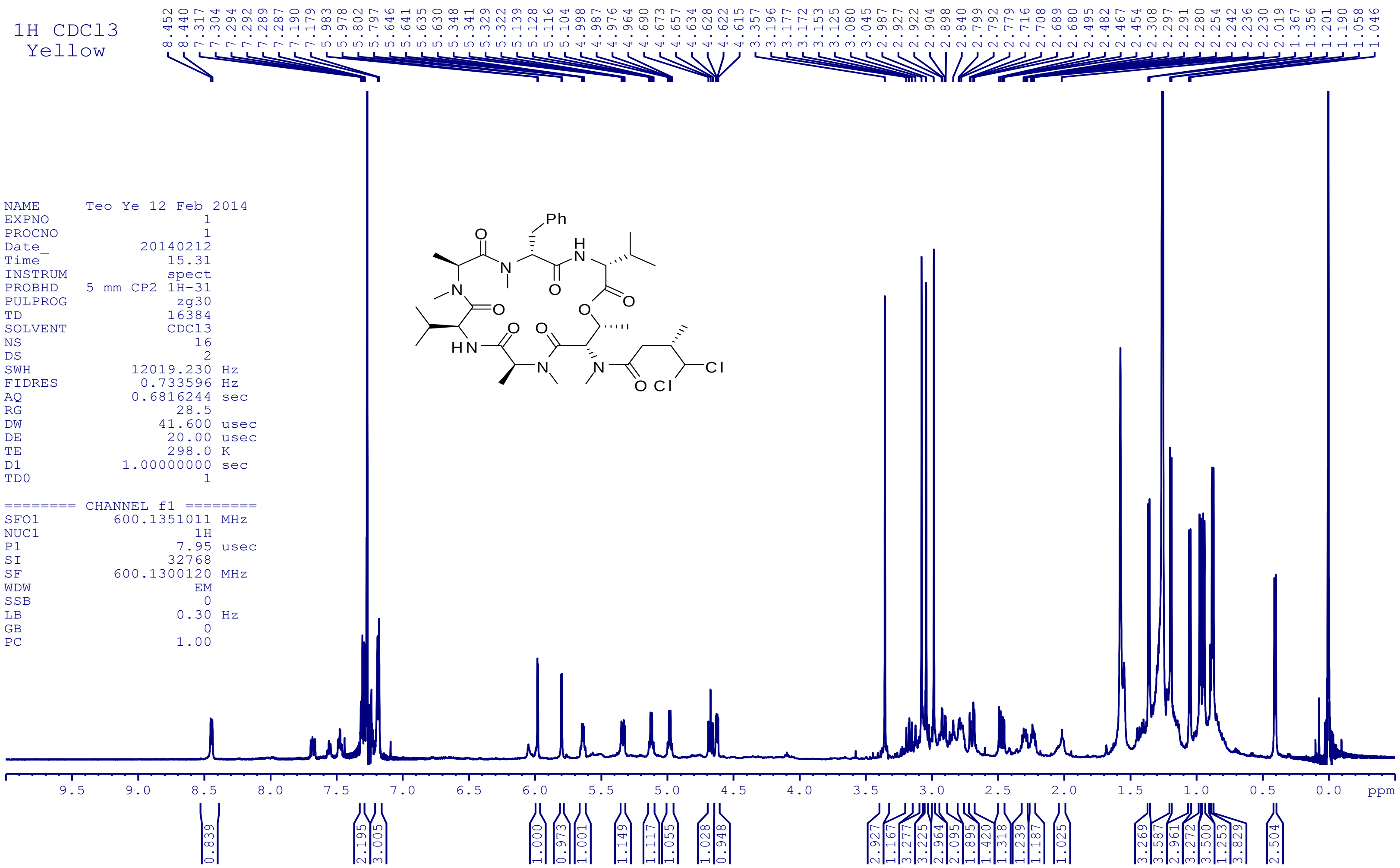

Figure S17. ^{1}H NMR of itralamide B **1c**.

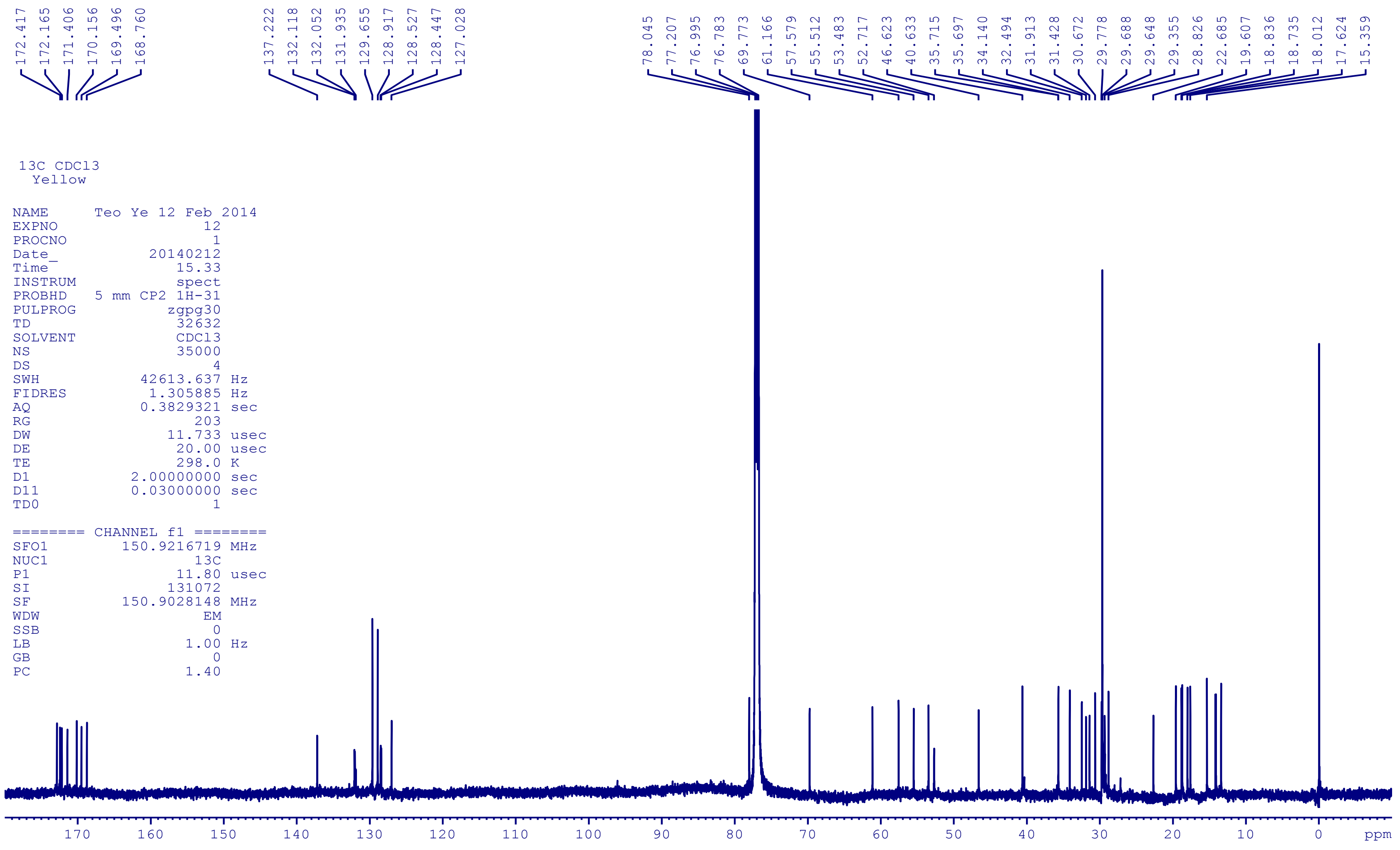

Figure S18. ^{13}C NMR of itralamide B **1c**.

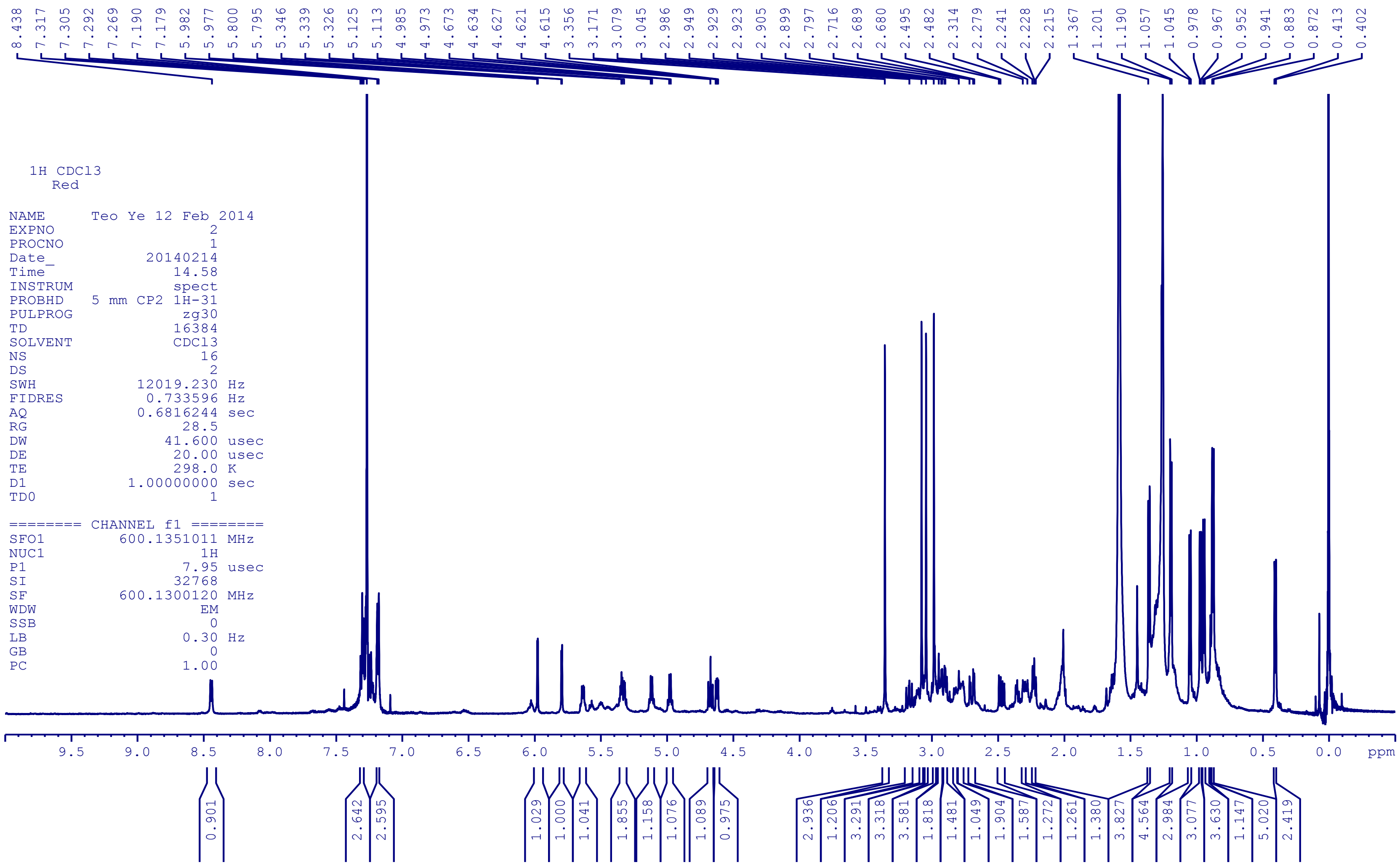

Figure S19. ^{1}H NMR of itralamide B **1d**.

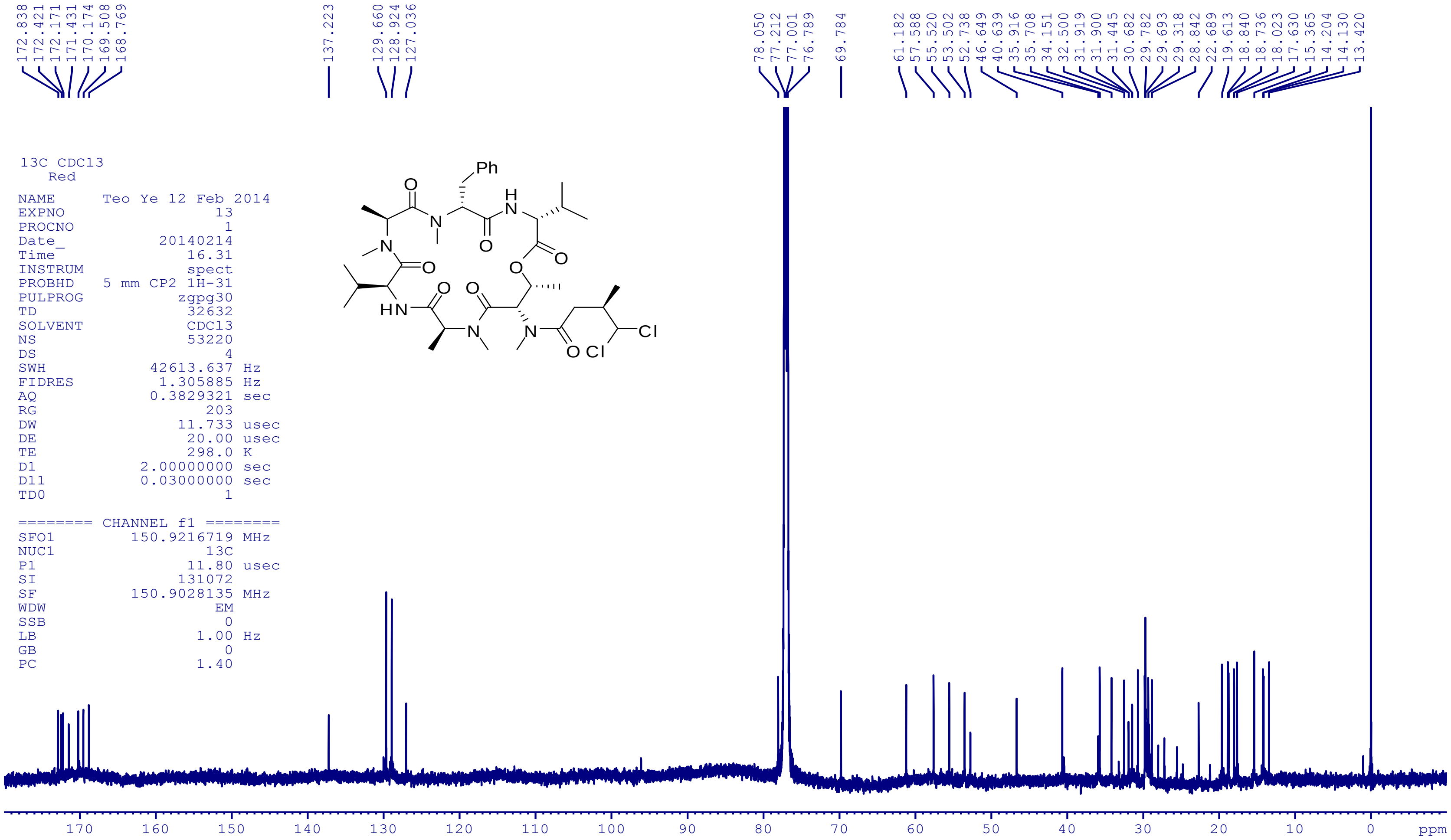

Figure S20. ^{13}C NMR of itralamide B **1d**.

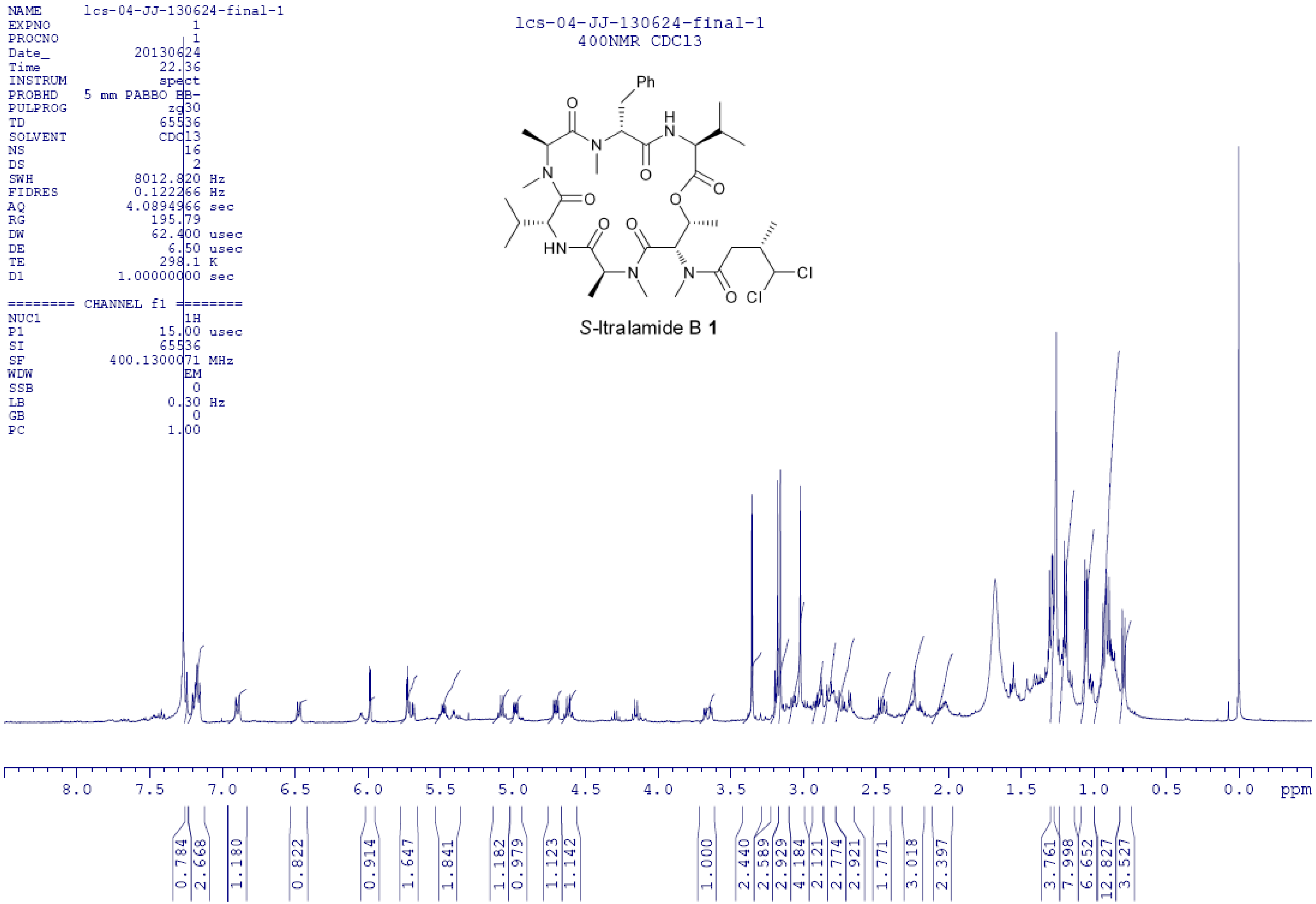

Figure S21. ^{1}H NMR of itralamide B **1a**.

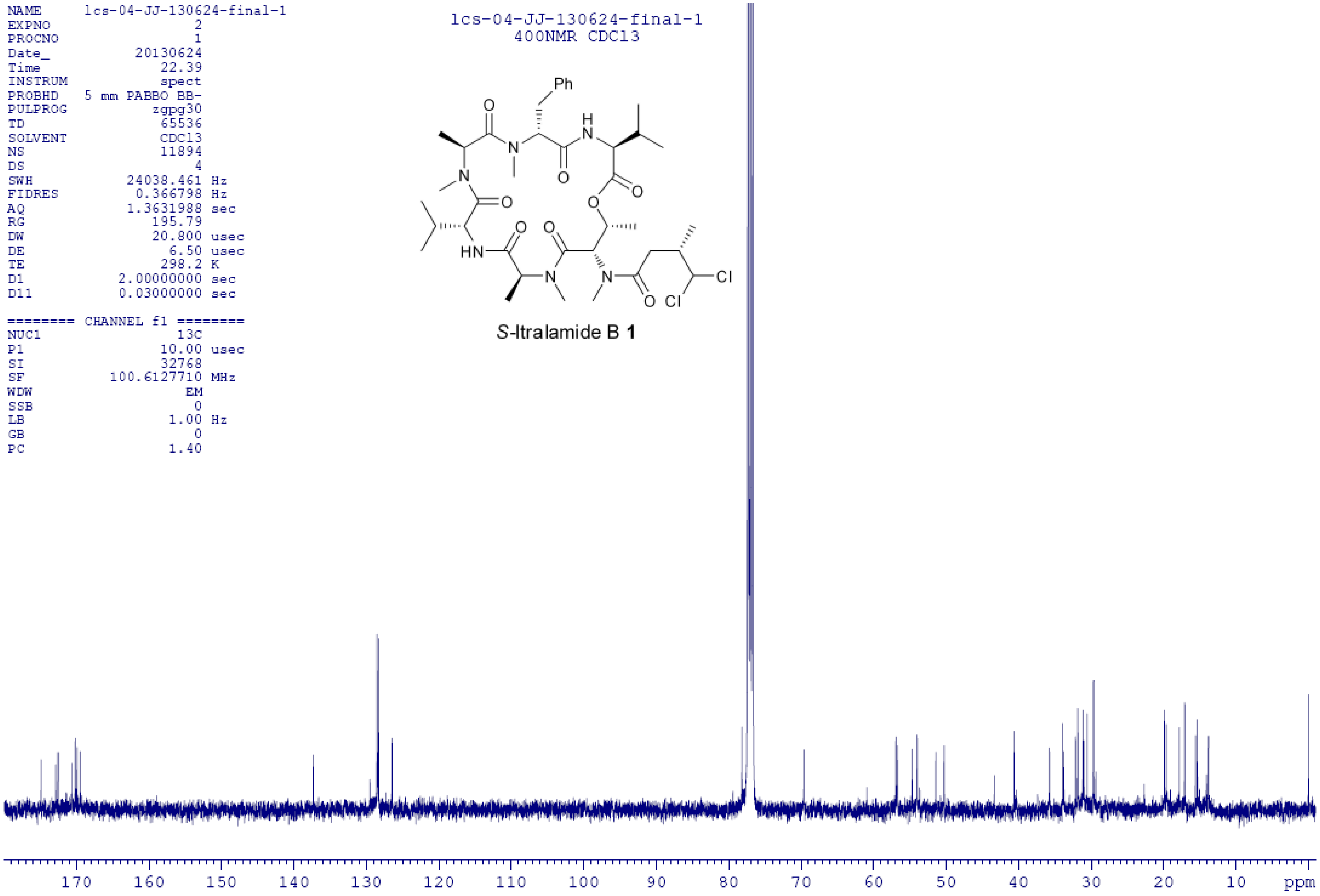

Figure S22. ^{13}C NMR of itralamide B **1a**.

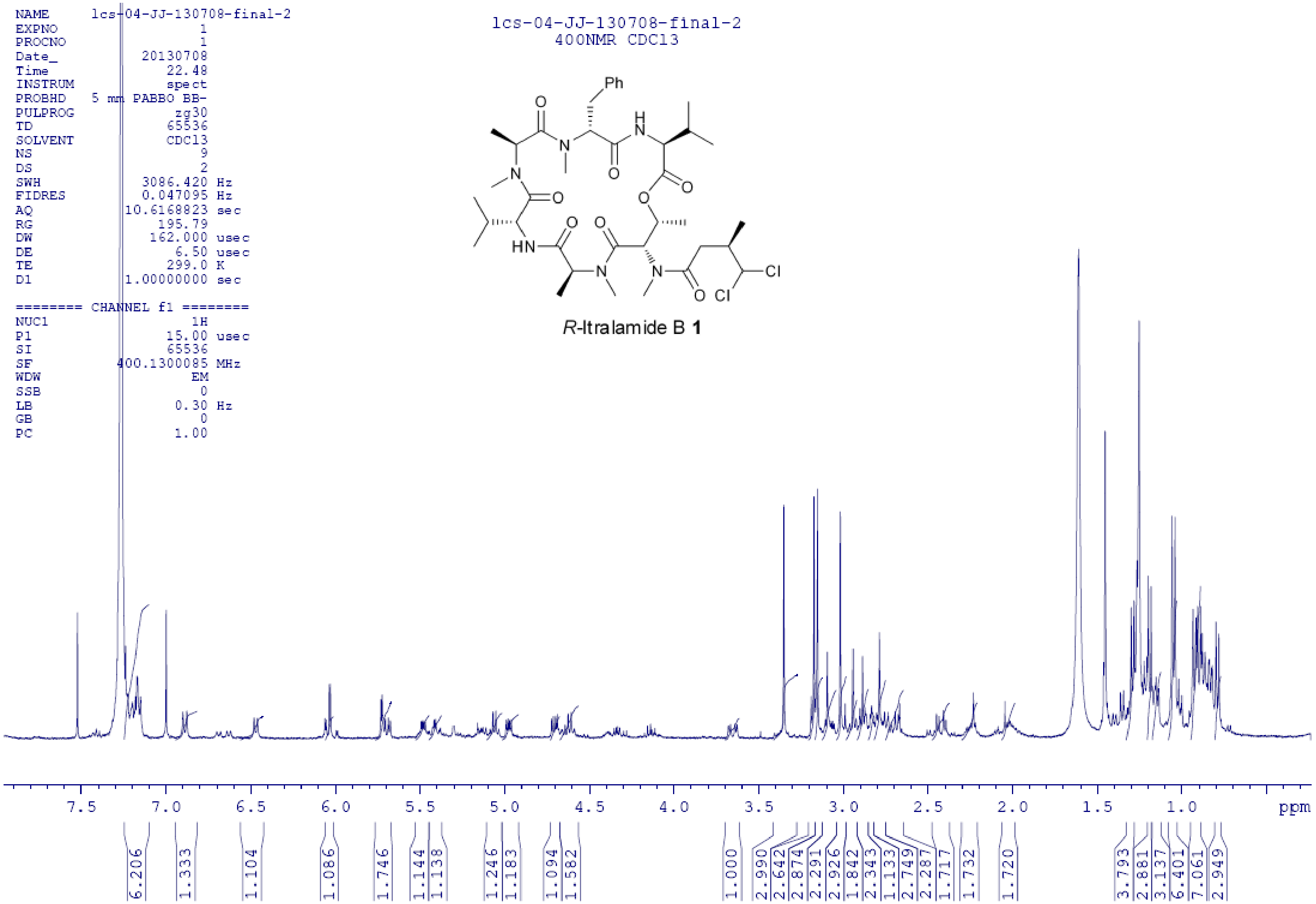

Figure S23. ^{1}H NMR of itralamide B **1b**.

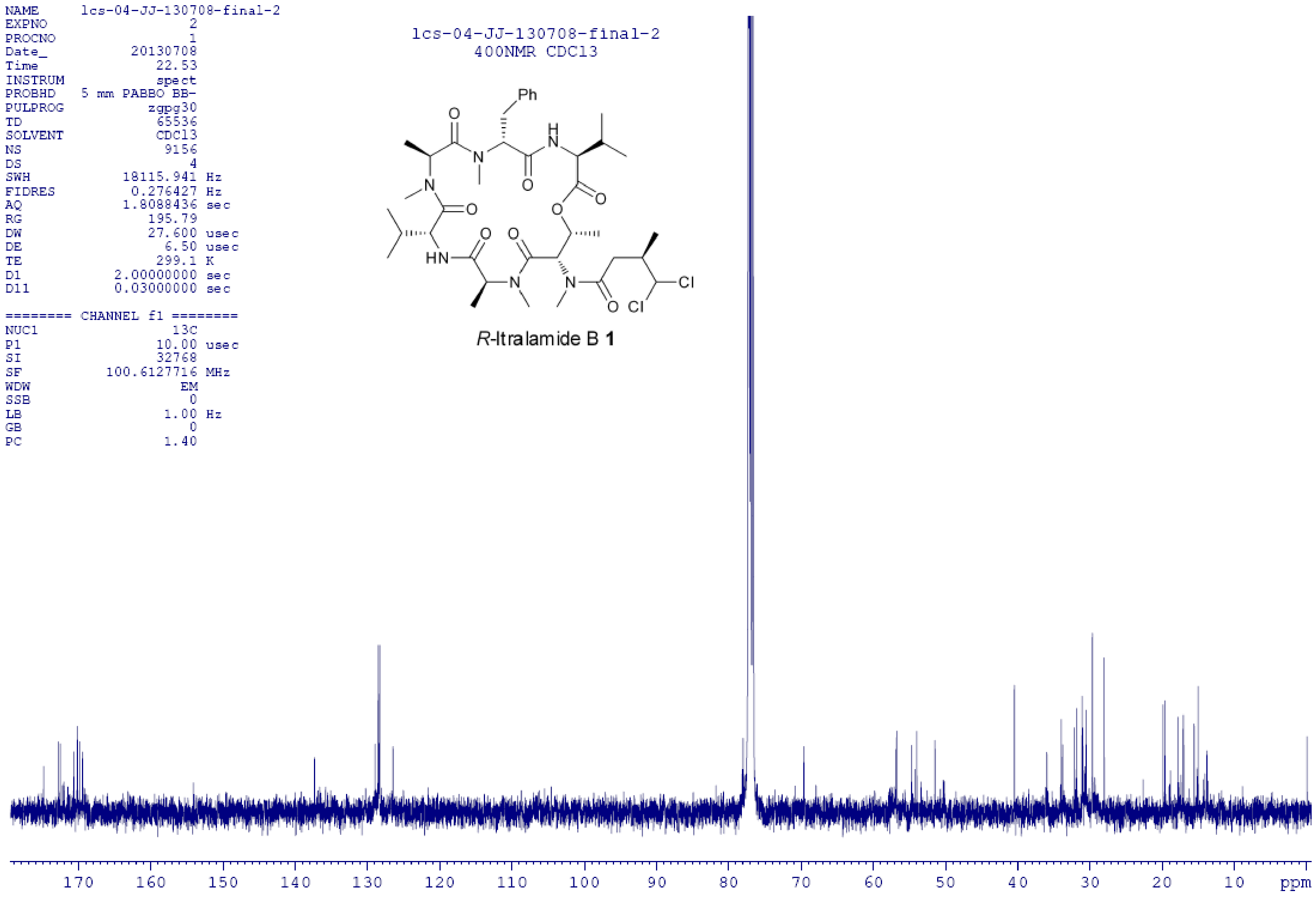

Figure S24. ^{13}C NMR of itralamide B **1b**.

Table S1. Comparison of ^{13}C NMR data of natural itralamide B with synthetic samples.

Carbon No.	$\delta_{Natural\ product}$	δ_{1a}	δ_{NP}-δ_{1a}	δ_{1b}	δ_{NP}-δ_{1b}	δ_{1c}	δ_{NP}-δ_{1c}	δ_{1d}	δ_{NP}-δ_{1d}
2	173.9	174.89	−0.99	174.88	−0.98	172.83	1.07	172.84	1.06
3	56.6	56.75	−0.15	56.75	−0.15	57.6	−1	57.59	−0.99
3a	32	32.15	−0.15	32.18	−0.18	32.49	−0.49	32.5	−0.5
3b	20.5	19.63	0.87	19.63	0.87	19.61	0.89	19.61	0.89
3c	16.6	17.1	−0.5	17.09	−0.49	17.62	−1.02	17.63	−1.03
5	-	169.47	-	169.51	-	168.76	-	168.77	-
6	56.7	56.92	−0.22	56.88	−0.18	61.17	−4.47	61.18	−4.48
6a	33.4	33.77	−0.37	33.78	−0.38	34.14`	−0.74	34.15	−0.75
6b	137.5	137.35	0.15	137.37	0.13	137.22	0.28	137.22	0.28
6c/6c'	128.5	128.54	−0.04	128.55	−0.05	129.66	−1.16	129.66	−1.16
6d/6d'	128.3	128.34	−0.04	128.34	−0.04	128.92	−0.62	128.92	−0.62
6e	126.3	126.48	−0.18	126.48	−0.18	127.03	−0.73	127.04	−0.74
7a	31.5	31	0.5	31.02	0.48	30.67	0.83	30.68	0.82
8	172.7	172.84	−0.14	172.83	−0.13	172.42	0.28	172.42	0.28
9	51.6	51.43	0.17	51.47	0.13	46.62	4.98	46.65	4.95
9a	13	13.82	−0.82	13.83	−0.83	13.43	−0.43	13.42	−0.42
10a	31.6	31.12	0.48	31.12	0.48	31.43	0.17	31.45	0.15
11	172.1	170.65	1.45	170.68	1.42	171.41	0.69	171.43	0.67
12	54.9	54.69	0.21	54.75	0.15	55.51	−0.61	55.52	−0.62
12a	30.9	30.54	0.36	30.55	0.35	29.78	1.12	29.69	1.21
12b	20.8	19.9	0.9	19.89	0.91	22.69	−1.89	22.69	−1.89
12c	16.3	15.61	0.69	17.07	−0.77	14.21	2.09	14.2	2.1
14	170.4	170.17	0.23	170.17	0.23	170.16	0.24	170.17	0.23
15	53.5	53.98	−0.48	53.99	−0.49	52.72	0.78	52.74	0.76
15a	13.5	14.1	−0.6	15.05	−1.55	14.13	−0.63	14.13	−0.63

Table S1. *Cont.*

16a	31.7	31.83	−0.13	31.85	−0.15	31.91	−0.21	31.92	−0.22
17	169.9	169.9	0	169.85	0.05	169.5	0.4	169.51	0.39
18	54.8	54.68	0.12	54.22	0.58	53.48	1.32	53.5	1.3
18a	68.8	69.62	−0.82	69.64	−0.84	69.77	−0.97	59.78	−0.98
18b	17.7	17.82	−0.12	17.82	−0.12	18.01	−0.31	18.02	−0.32
19a	34.1	33.92	0.18	33.97	0.13	35.7	−1.6	35.71	−1.61
20	172.6	172.53	0.07	172.56	0.04	172.17	0.43	172.17	0.43
21	35.8	35.75	0.05	35.98	−0.18	35.72	0.08	35.92	−0.12
22	40.5	40.65	−0.15	40.45	0.05	40.63	−0.13	40.64	−0.14
22a	15.1	15.35	−0.25	15.6	−0.5	15.37	−0.27	15.36	−0.26
23	77.8	78.18	−0.38	78.02	−0.22	78.05	−0.25	78.05	−0.25

© 2015 by the authors; licensee MDPI, Basel, Switzerland. This article is an open access article distributed under the terms and conditions of the Creative Commons Attribution license (http://creativecommons.org/licenses/by/4.0/).
